# Ultrasensitive digital quantification of cytokines and bacteria predicts septic shock outcomes

**DOI:** 10.1038/s41467-020-16124-9

**Published:** 2020-05-25

**Authors:** M. Fatih Abasıyanık, Krysta Wolfe, Hoang Van Phan, Jing Lin, Bharathi Laxman, Steven R. White, Philip A. Verhoef, Gökhan M. Mutlu, Bhakti Patel, Savaş Tay

**Affiliations:** 1 Pritzker School of Molecular Engineering, The University of Chicago, Chicago, IL 60637 USA; 2 Institute for Genomics and Systems Biology, The University of Chicago, Chicago, IL 60637 USA; 3 Department of Medicine, Section of Pulmonary/Critical Care, The University of Chicago, Chicago, IL 60637 USA; 4 Center for Integrated Health Research, Kaiser Permanente Hawaii, Honolulu, HI 96819 USA

**Keywords:** Assay systems, Molecular medicine, Sepsis

## Abstract

Quantification of pathogen and host biomarkers is essential for the diagnosis, monitoring, and treatment of infectious diseases. Here, we demonstrate sensitive and rapid quantification of bacterial load and cytokines from human biological samples to generate actionable hypotheses. Our digital assay measures IL-6 and TNF-*α* proteins, gram-negative (GN) and gram-positive (GP) bacterial DNA, and the antibiotic-resistance gene *bla*_TEM_ with femtomolar sensitivity. We use our method to characterize bronchoalveolar lavage fluid from patients with asthma, and find elevated GN bacteria and IL-6 levels compared to healthy subjects. We then analyze plasma from patients with septic shock and find that increasing levels of IL-6 and *bla*_TEM_ are associated with mortality, while decreasing IL-6 levels are associated with recovery. Surprisingly, lower GN bacteria levels are associated with higher probability of death. Applying decision-tree analysis to our measurements, we are able to predict mortality and rate of recovery from septic shock with over 90% accuracy.

## Abstract (web summary)

Ultrasensitive methods for detection of biomarkers for infectious disease are needed for diagnosing, monitoring and targeting treatment. Here the authors develop a digital assay for inflammatory markers, bacterial DNA and antibotic-resistance genes and apply it to characterise asthma patients and predict mortality from septic shock.

## Introduction

Over 3.8 million hospitalizations in 2011 in the United States involved infectious pathogens, which were nearly twice as high as in 1997. Sepsis and pneumonia accounted for over 2 million of these hospitalizations^1^. Sepsis is a dysregulated host response to an infection, leading to life-threatening organ dysfunction. Septic shock is a subset of sepsis characterized by circulatory dysfunction, leading to tissue hypoperfusion and a higher risk of death^2^. While advances in early recognition and supportive care have improved outcomes, mortality for sepsis and its most severe manifestation, septic shock, remains unacceptably high^3–5^. Despite years of research, diagnosis and prognostication of sepsis and septic shock predominantly rely on clinical criteria. Numerous biomarkers have been studied, but in isolation, none of them have the accuracy necessary to be used in clinical practice^6^. In order to identify patients most likely to benefit from targeted interventions, new tools are needed that allow accurate characterization of the diverse pathogen and host factors that shape outcomes for patients with sepsis and septic shock.

Pathogens, their toxins, and their nucleic acids can trigger the dysregulated host response of sepsis. Early recognition of the infectious trigger and treatment with appropriate antibiotics is critical to improving outcomes in sepsis^7^. However, this often relies on clinical intuition, nonspecific clinical markers, or screening tools with poor specificity^8^. Every hour delay in administration of appropriate antibiotics results in a 4% increase in odds of death^7^. Strategies to improve sepsis and septic shock outcomes must include the rapid identification of infectious pathogens and their antibiotic-resistance patterns, which typically depends on culturing of relevant biological samples. Unfortunately, culture-based diagnoses are slow (requiring 1–5 days), labor-intensive, have poor resolution, and are often nonquantitative. New tools that detect infection directly and can be used to monitor the pathogen’s response to treatment longitudinally could dramatically improve the timeliness and specificity of treatment in infectious diseases^9,10^.

Equally important to identification of the inciting pathogen in sepsis is the characterization of the host immune response. Despite decades of research, there remain no targeted therapies for sepsis or septic shock due in part to the wide heterogeneity of this disease^11,12^. The inability to accurately break down this heterogeneity at the bedside is a major barrier to achieving targeted and precise therapies. The immune response to sepsis is highly complex, but typically involves a proinflammatory phase, with secretion of cytokines such as TNF-α and IL-6^13–15^. Therefore, the ideal approach to disease monitoring and characterization combines tracking the most common pathogens that trigger sepsis and septic shock (typically GN and GP bacteria)^11,16,17^ with monitoring of host inflammatory cytokine levels.

Longitudinal biomarker analysis requires rapid sample processing, the ability to detect small differences between samples (i.e., high resolution), and low concentrations of analytes (i.e., low limit of detection), while using the least amount of biological samples possible. A high resolution is required to determine whether different readings at different time points correspond to true biological differences, or measurement noise^10^. However, most commercially available molecular assays for cytokine quantification are based on ELISA, which typically have poor sensitivity, are not suitable for simultaneous measurement with other types of biomarkers (such as bacterial DNAs and plasmids), and require large volumes of sample. Proximity ligation assay (PLA) can analyze low-abundant proteins^18^ by amplifying signals from antibody–antigen-binding events, making it suitable for developing a multiplex assay for quantifying protein and nucleic acid targets at the same time. Nevertheless, conventional PLA relies on real-time PCR (qPCR) readout, which has high measurement noise and low resolution^19^.

Here, we report the development of highly sensitive, rapid, and highly specific digital proximity ligation assays (dPLA) for quantifying both nucleic acid and protein markers in infectious diseases. By using droplet digital-PCR (ddPCR) readout in the PLA protocol, we enabled simultaneous measurement of GN- and GP-specific 16S rRNA genes (which reflect the abundance of all GN and GP bacteria in the patient samples), and the *bla*_TEM_ gene (which induces resistance to the β-lactam antibiotics) together with IL-6 and TNF-α protein levels in the same patient sample. A major advantage of our digital amplification method is its ability to quantify very small changes in the concentration of these molecules. ddPCR has a resolution of a single-DNA molecule in samples^20^, and we were able to achieve sub-femtomolar resolution for protein targets.

To demonstrate the potential of our approach, we first used it to analyze bronchoalveolar lavage fluid (BALF) samples from patients with mild-to-severe asthma, and found that patients with asthma had higher levels of GN bacteria and IL-6 than healthy control subjects. We further used our assays to longitudinally characterize plasma samples from patients with septic shock, revealing several molecular features associated with recovery or death. Our analyses showed that temporal changes in several biomarkers, and not the absolute concentrations, are reliable predictors of patient outcomes. We applied decision tree analysis to predict patient mortality and the rate of recovery from septic shock with over 90% accuracy using measurements from our method.

## Results

### Ultrasensitive digital quantification of TNF-α and IL-6

**Fig. 1:**
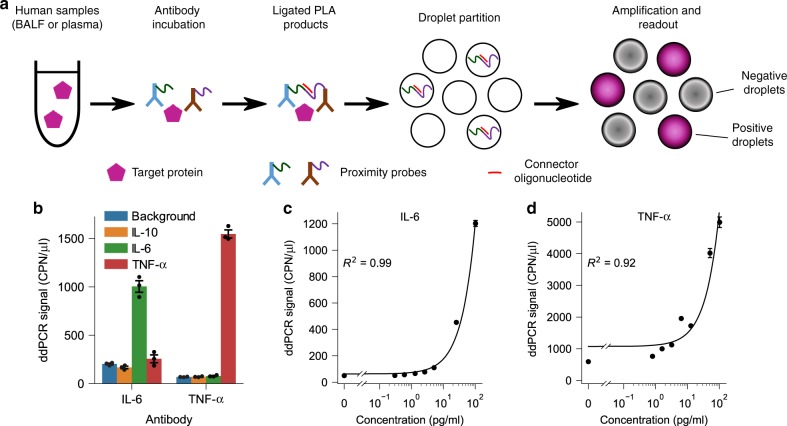
Ultrasensitive quantification of human proteins using digital PLA. **a** In digital PLA, each detected protein molecule is bound by a pair of DNA oligonucleotide-conjugated antibodies (i.e., proximity probes), the oligonucleotides are ligated with the help of a connector, and the ligated products are quantified by droplet digital-PCR (ddPCR). **b** The specificity of dPLA was tested by using IL-6 and TNF-α antibodies against IL-6, TNF-α and IL-10 spike-in standards. Only IL-6 spike-in produced a high signal with IL-6 antibody, and only TNF-α produced a high signal with TNF-α antibody. **c**, **d** Calibration curves for IL-6 (**c**) and TNF-α (**d**) spiked in with healthy human plasma. The solid lines in (**c**, **d**) are the linear regression of the calibration curves. Data are presented as mean ± s.e.m., and each concentration has three technical replicates.

In conventional PLA, a pair of DNA oligonucleotide-conjugated antibodies bind to the target protein, the oligonucleotides become ligated, and the ligated DNA molecules are amplified and quantified by qPCR. PLA has been used in a variety of applications, including protein quantification and detection, pathogen detection, and detection of protein–protein interactions^21^. Despite its inherent advantages, conventional PLA has limited resolution because the qPCR readout can only reliably detect changes of twofold or higher. We recently developed digital PLA (dPLA), which combines ddPCR and PLA for accurate and sensitive characterization of proteins^19^ (Fig. 1). The advantages of using ddPCR as a readout include absolute quantification and low measurement noise, and thereby improving the limit of detection (LOD), sensitivity, and resolution of molecular assays^22^. In ddPCR, DNA molecules are distributed into nanoliter aqueous droplets in oil in limiting dilution, such that each droplet contains either zero or one molecule^23^. PCR amplification occurs inside droplets, and subsequent counting of positive droplets allows sensitive and absolute quantification of nucleic acids (Fig. 1a; Supplementary Fig. 1).

**Fig. 2:**
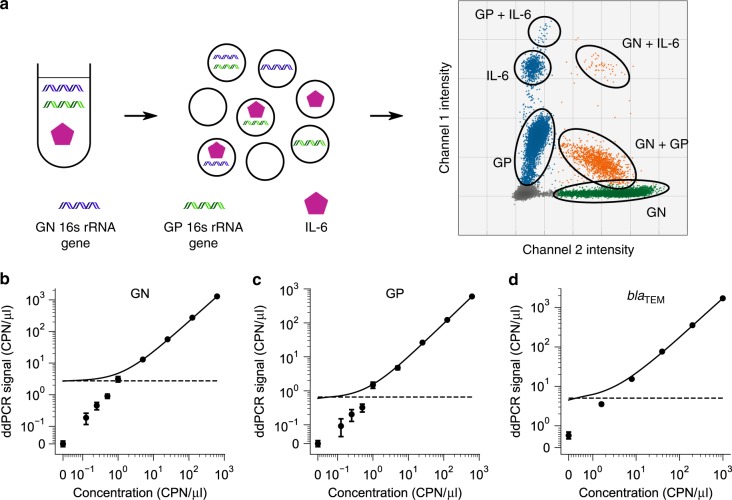
Ultrasensitive multiplexed quantification of nucleic acid and protein biomarkers. **a** We developed a triplex assay where two DNA signals and one protein signal can be detected in the same droplet digital-PCR reaction. The operating principle of the triplex digital assay is shown in **a**. Three biomarkers (GN, GP bacteria, and IL-6) can be measured simultaneously by multiplexing the two fluorescence channels and the fluorescence intensity of positive droplets (as is the case for GP and IL-6 in the right panel). **b**–**d** Calibration curves for quantification of GN, GP bacterial, and the bacterial antibiotic-resistant gene *bla*_TEM_, respectively. CPN/μl stands for the absolute DNA molecule copy number per microliter. In (**b**–**d)**, the solid lines are the linear regression of the calibration curve, and the horizontal dashed lines indicate the LOD of the assay. Note the scale of the axes in (**b**, **c**): it is linear between 0 and 0.1, and logarithmic from 0.1 and higher. Note the scale of the axes in (**d**): it is linear between 0 and 1, and logarithmic from 1 and higher. Data in (**b**–**d**) are presented as mean ± s.e.m., and each concentration has three technical replicates.

**Fig. 3:**
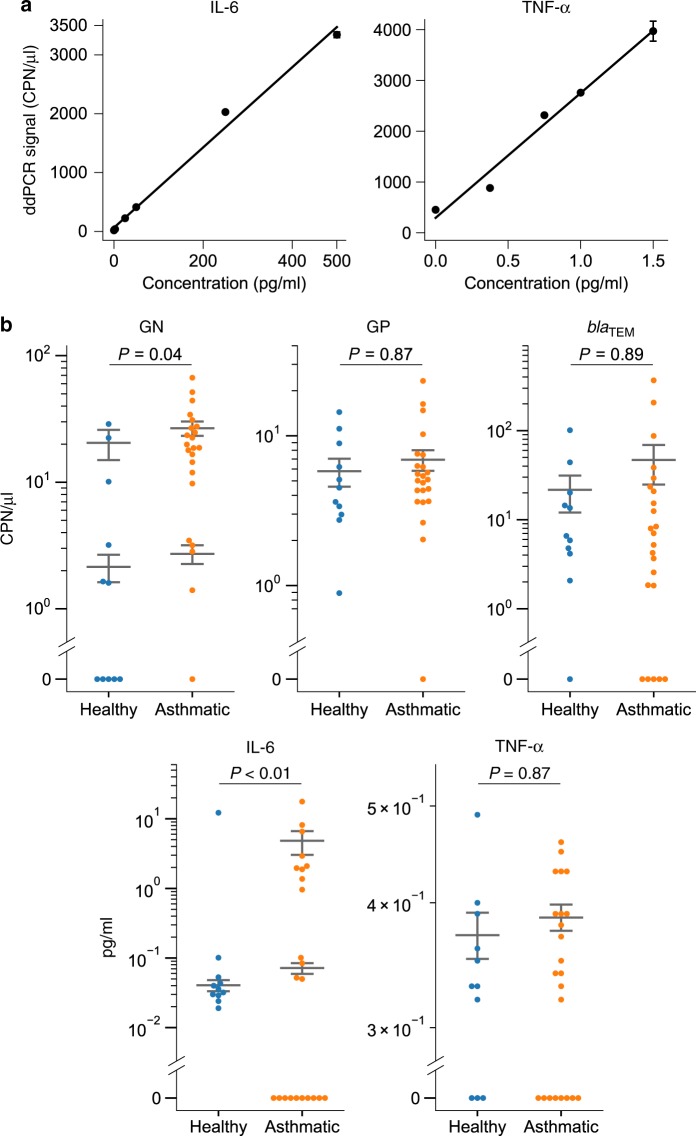
Molecular markers for asthmatic inflammation. **a** Example digital PLA calibration curves of IL-6 and TNF-α in human BALF samples. Data in (**a**) are presented as mean ± s.e.m., and each concentration has three technical replicates. **b** The measured concentrations of GN and GP bacteria, *bla*_TEM_ genes, IL-6, and TNF-α proteins in the healthy subjects (*n* = 11) and patients with asthma (*n* = 23). The gray lines in (**b**) indicate the mean ± s.e.m. of the populations (or subpopulations) of detectable samples within each group. (**b**) Two-sided two-part statistical test^62,63^ with Benjamini–Hochberg correction.

In this study, we were motivated by the unmet need for methods that can accurately and sensitively characterize inflammatory cytokines together with bacterial load in human biological fluids. Protein analysis in human biological samples is challenging since the cross-reactivity of antibodies can substantially increase the background signals^24^. Further, various inhibitory factors in human plasma can undermine several key steps in PLA and ddPCR reactions. We have overcome these challenges to develop new dPLA assays for IL-6 and TNF-α, and optimized the PLA and ddPCR assay conditions for quantification of these biomarkers in human samples with high precision, resolution, and reproducibility (Fig. 2; Supplementary Figs. 2–3). For IL-6 and TNF-α quantification in BALF samples, we achieved sample LODs of ~0.02 pg/ml (0.73 fM) and 0.08 pg/ml (4.33 fM), respectively (Fig. 3a; Supplementary Table 1). We also confirmed the specificity of our dPLA assay by using IL-6 and TNF-α antibodies on IL-6, TNF-α, and IL-10 standards (Fig. 1b). We found that our assay displayed excellent specificity: true signals were one to two orders of magnitude as high as that of nonspecific, cross-reactive signals, which were themselves as low as the background signals.

To test whether our dPLA assay would be compatible with human plasma samples, we first spiked in IL-6 standards with chicken plasma as the representative medium that mimics the complex composition of human plasma while having minimal antibody cross-reactivity^25^. We found that the commercially available dilution buffer, SDB-II (see “Methods”), showed good performance with chicken plasma (Supplementary Fig. 3f) across five orders of magnitude (Supplementary Fig. 3d). Next, we tested the buffer SDB-II with healthy human plasma, and found that our IL-6 dPLA was indeed compatible with human plasma samples (Fig. 1c), with a sample LOD of 0.84 pg/ml (40 fM) (Supplementary Table 2). However, SDB-II was not compatible with TNF-α measurements in chicken plasma. This is probably because TNF-α is very sensitive to storage and buffer conditions, and degrades rapidly^26–28^. Therefore, we developed a custom homemade buffer (HMB) based on the Nong’s buffer^29^ for TNF-α quantification in plasma samples (Fig. 1d). To compare the performance of our dPLA assays against commercial ELISA kits, we performed IL-6 and TNF-α quantification in undiluted SDB-II and HMB buffers, respectively. We found that our sample LODs for IL-6 and TNF-α were as low as 0.09 pg/ml and 0.08 pg/ml (4.12 fM and 4.58 fM), respectively. These values are better than many available ELISA kits (Supplementary Tables 3 and 4).

### Ultrasensitive digital quantification of bacterial markers

For ddPCR quantification of bacterial DNA, we used a pair of universal forward and reverse primers that target regions of the 16S rRNA gene that are common to both GN and GP bacteria, and a fluorescent hydrolysis probe targeting the region specific to either GN or GP bacteria^30^ (Supplementary Table 5). We note that our ddPCR protocols detect both pathogenic bacteria and endogenous microbiota that may be present in the patient samples. We validated the specificity of the primers/probe (Supplementary Fig. 4a), optimized the primer concentration (Supplementary Fig. 4d), and estimated the LOD of our bacteria quantification assay to be 1.76 CFU/μl and 12.19 CFU/μl for GN and GP bacteria, respectively (Supplementary Fig. 4b, c). The LOD of the bacterial DNA assay can be further improved by preconcentrating the human samples during the DNA extraction step.

To further benchmark our ddPCR assay, we quantified DNA standards from gBlock fragments (Supplementary Fig. 5a, b). We found that GN, GP, and *bla*_TEM_ quantification achieved LODs of 0.06, 0.06, and 0.9 copy number per μl (CPN/μl), respectively, which corresponded to 0.09, 0.09, and 1.57 aM concentration (Fig. 2b–d). All measurements showed a high degree of linearity, even at the concentrations close to the LOD. The coefficient of variations (CVs) for these measurements could be as low as 1.3%. This high precision also enabled ddPCR to be much more sensitive than qPCR, especially for GP quantification (Supplementary Fig. 5c). Last, ddPCR quantification showed extremely low technical variability: repeated measurements of the same gBlock samples were almost equal across 100 days (Supplementary Fig. 5d).

### Joint quantification of host cytokines and bacterial markers

Performing a separate ddPCR reaction for each sample increases the labor and costs, and introduces measurement variability. We optimized a duplex ddPCR assay for measuring GN and GP, and a triplex ddPCR assay for measuring GN, GP, and *bla*_TEM_ genes from the same sample simultaneously (Supplementary Fig. 5a, b). Triplex and duplex measurements of GN and GP bacteria were also confirmed to agree with each other (Supplementary Fig. 6).

We also improved the dPLA assay so that the concentrations of IL-6, GN, and GP-specific 16S rRNA genes could be quantified from the same BALF sample, and in the same ddPCR reaction. To this end, we used two different fluorescent probes in the same ddPCR droplet, and different fluorescent-intensity levels in the positive droplets to allow further identification of signals (Fig. 2a). The LOD levels we achieved for this triplex assay for IL-6, GP, and GN bacteria were 0.1 pg/ml (4.76 fM), 6.8 CPN/μl (11.29 aM), and 10.8 CPN/μl (17.93 aM), respectively.

### GN bacteria and IL-6 correlate with airway inflammation

Characterization of BALF is used in the diagnosis of lung diseases such as pneumonia, lung cancer, and interstitial lung diseases^31,32^. We used our method to quantify GN, GP bacteria, *bla*_TEM_ gene, IL-6, and TNF-α in human BALF samples from 11 healthy subjects and 23 subjects with asthma. In both healthy and asthmatic groups, a proportion of the samples had nondetectable levels of either proteins or DNA biomarkers (Fig. 3b; Supplementary Fig. 7). GN and GP measurements of healthy individuals’ plasma samples showed background-level signals (Supplementary Fig. 8). We observed large differences in GN bacterial load in asthmatic patients: almost all patients had high GN levels, compared with only 55% of the healthy individuals (Supplementary Fig. 7). In addition, the GN levels of both asthmatic and healthy individuals showed two nonzero subpopulations, one at ~20 CPN/μl, and one at ~2 CPN/μl. These results are in agreement with a recent study that found an increase in GN bacteria in sputum samples from patients with asthma^33^.

TNF-α concentrations in all BALF samples were uniformly low (below 0.5 pg/ml, which was barely above the LOD of the assay). On the other hand, IL-6 levels in asthmatic patients were significantly higher than most healthy individuals (Fig. 3b; Supplementary Fig. 7). One healthy individual was an outlier: this person’s IL-6 level was slightly higher than 10 pg/ml, which was two orders of magnitude as high as that of the other healthy samples. Our results agree with a study by Ilmarinen et al., which suggested that elevated IL-6 levels might reflect a proinflammatory state of the lung, and that IL-6 expression could serve as a biomarker for asthma^34^.

### Biomarker levels rapidly change in septic shock patients

**Fig. 4:**
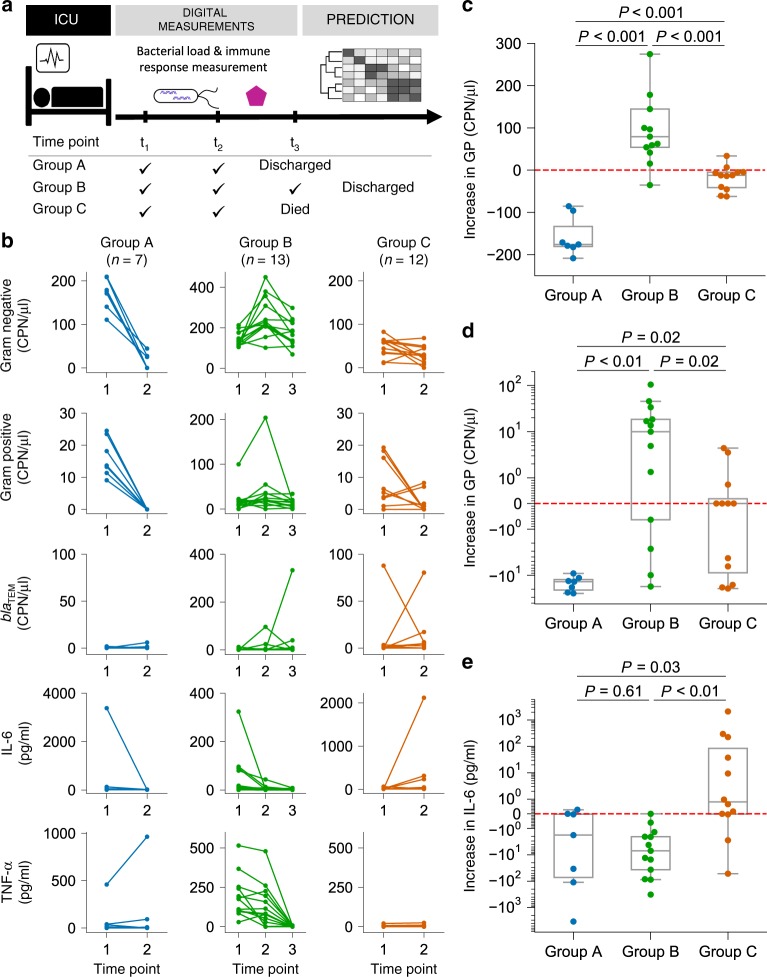
Correlation between septic shock outcomes and changes in GN bacteria, IL-6 protein, and *bla*_TEM_. **a** Plasma was collected from patients with septic shock in the ICU, and measured by our digital PLA/digital PCR protocol at different time points to quantify cytokines and bacterial DNA. Patients were classified into three groups by their outcomes, i.e., early recovery (group A) (*n* = 7), late recovery (group B) (*n* = 13), and death (group C) (*n* = 12). **b** Measurements of GN bacteria, GP bacteria, *bla*_TEM_, IL-6, and TNF-α in the three patient groups. **c**–**e** Temporal increase in (**c**) GN, (**d**) GP, and (**e**) IL-6 between time points *t*_1_ and *t*_2_. In (**c**–**e**), each box shows the lower and upper quartile values, the line inside the box shows the median, and the whiskers extend to the value 1.5× the interquartile range beyond the box. The red horizontal dashed lines indicate no detectable changes between the two time points. Note the scale of the y axes in (**d**, **e**): the scale is linear between −1 and 1, logarithmic otherwise, and is symmetric about 0. (**c**–**e**) Two-sided Mann–Whitney *U* test with Benjamini–Hochberg correction.

We applied our assays in plasma samples from patients with septic shock. Patients admitted to the intensive care unit (ICU) with septic shock had blood drawn at two time points: *t*_1_ = within the first 24 h of meeting the Sepsis-3 definition of septic shock^2^, and *t*_2_ = 24–48 h after shock diagnosis (Fig. 4a). The patients’ clinical characteristics are listed in Supplementary Tables 6 and 7. Three distinct clinical phenotypes were identified a priori. Group A patients (*n* = 7) had resolution of septic shock within 48 h of enrollment (as defined by no-longer-requiring vasopressors to maintain a mean arterial pressure greater than 65 mmHg), and were designated as early recovery. Group B patients (*n* = 13) required more than 48 h for shock resolution, and were designated as late recovery. Group B patients had a third blood sample drawn at an additional third time point, *t*_3_ = 24 h after resolution of shock. Group C patients (*n* = 12) never recovered from septic shock and died in the ICU. Plasma was collected and frozen at −80 °C. More information on the sample collection is available in the “Methods”.

We quantified the amount of GN and GP bacteria, *bla*_TEM_ level, IL-6, and TNF-α in plasma from patients with septic shock (Fig. 4b; Supplementary Fig. 9), and observed clear correlations between patient outcomes and the temporal changes of GN, GP, and IL-6. First, we confirmed that plasma samples from healthy individuals did not contain any detectable bacterial levels (Supplementary Fig. 8a, b). On the other hand, almost all septic shock patients had detectable levels of GN and GP bacteria at time point *t*_1_ (Fig. 4b). Further, all patients in group A (early recovery) had GN levels that decreased rapidly between time points *t*_1_ and *t*_2_ (average decrease was ~157 CPN/μl) (Fig. 4c). We also confirmed with qPCR that the GN levels at *t*_2_ in group A were significantly lower than those at *t*_1_ (Supplementary Fig. 8c). In contrast, the GN levels in all but one patient in the late-recovery group (group B) increased quickly (average increase was ~108 CPN/μl). Surprisingly, GN levels of patients who died (group C) decreased slightly by time point *t*_2_. The levels of GP bacteria in all patients in group A and the majority of group C decreased to a nondetectable level at time point *t*_2_ (Fig. 4b, d; Supplementary Fig. 8d). However, the GP bacteria level of group B patients remained at a moderate level across all three time points (their average levels varied between 13 and 36 CPN/μl). This was surprising because these patients ultimately recovered from septic shock while still having detectable levels of bacteria in their plasma.

We found that most patients had *bla*_TEM_ levels below the detection limit (Supplementary Fig. 9). One patient in group B, the late-recovery group, had a significant increase in *bla*_TEM_ between time points *t*_2_ and *t*_3_ (from 0 to over 300 CPN/μl, Fig. 4b). This patient’s culture drawn at the same time as the *t*_3_ sample was positive for β-lactam-producing bacteria. This highlights the strength of our digital assay: β-lactam resistance can be detected on the same day of blood collection, without having to wait several days for the blood culture results. Regarding TNF-α, group B had the highest average levels among the three groups at both time points *t*_1_ and *t*_2_ (Fig. 4b). However, TNF-α levels of all patients in that group dropped drastically to below 10 pg/ml by time point *t*_3_, which may reflect the resolution of septic shock. Patients in group C showed a very small amount of TNF-α expression. Previous single-cell studies of cytokine secretion indicate a pronounced peak at 4–8 h, followed by quickly diminishing TNF-α secretion^35,36^. The patients in group C may have progressed beyond this initial phase of cytokine secretion by the time their TNF-α levels were measured.

### Changes in GN bacteria and IL-6 correlate with mortality

We found that temporal changes in IL-6 plasma levels strongly correlated with patient mortality (Fig. 4e). Among patients with a detectable increase in IL-6, 89% belonged to group C (the nonsurviving group). On the other hand, among patients with a decrease in IL-6, 85% belonged to either group A or B. Therefore, a drastic decrease or increase in IL-6 levels could potentially serve as a marker for patient survival or death, respectively. On the other hand, the absolute levels of IL-6 did not correlate with patient outcomes. Our results highlight the importance of dynamic, temporally resolved measurements in biomarker discovery, and their implications for monitoring and personalized therapy.

### Accurate prediction of septic shock patient outcomes

**Fig. 5:**
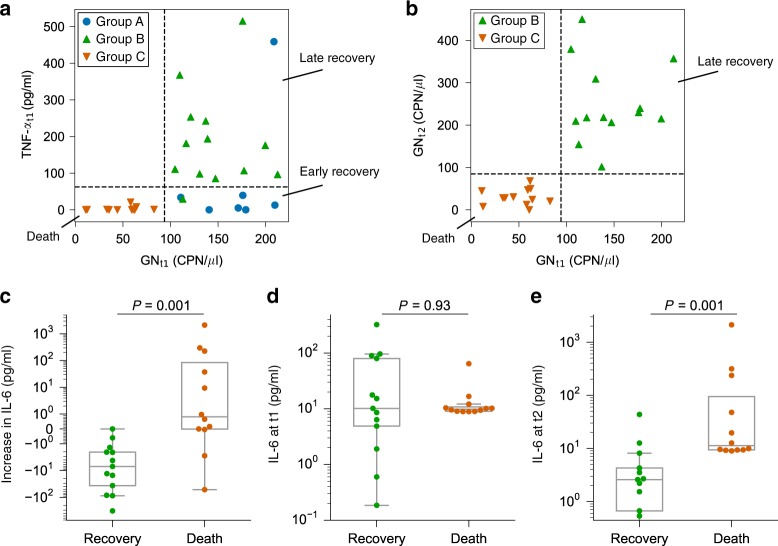
Accurate classification of septic shock patient outcomes. **a** Decision-tree classification shows that the patients can be classified with excellent accuracy using GN bacteria load and TNF-α level at time point *t*_1_ (the dashed lines represent the decision-making thresholds). **b** The level of GN bacteria at time points *t*_1_ and *t*_2_ in groups B and C also showed distinct separation thresholds (dashed lines). **c**–**e** Temporal changes of IL-6 (**c**), and the IL-6 levels at time point *t*_1_ (**d**) and time point *t*_2_ (**e**) of the patients who recovered (group B) (*n* = 13) or died (group C) (*n* = 12) from septic shock. In (**a**), the classification accuracies for groups A (*n* = 7), B (*n* = 13), and C (*n* = 12) are 85.71%, 92.31%, and 100%, respectively. Note that the scale of the *y* axis in (**c**) is symmetric and logarithmic. (**c**–**e**) Two-sided Mann–Whitney *U* test with Benjamini–Hochberg correction.

To find quantifiable relationships between our molecular measurements and patient outcomes, we classified patient outcomes using machine learning. We used the decision-tree classifier, which has the advantages of producing decision-making rules that are visible and simple to understand, while the number of computational layers can be controlled to avoid overfitting. By training the classifier with only *t*_1_ measurements, we found that patients could be classified into the three groups with high accuracy based on a threshold level of GN bacteria and TNF-α at the first time point (Fig. 5a). Here, only one patient in group A and one patient in group B were misclassified. This suggests that GN bacteria and TNF-α are effective and early markers that predict septic shock outcomes.

We also analyzed five other well-known sepsis markers across all available time points: procalcitonin, C-reactive protein (CRP), granulocyte colony-stimulating factor (G-CSF), IL-1β, and IL-8, as measured by a commercial bead-based immunoassay (see “Methods”). We found that none of these five additional markers exhibited a threshold level that differentiated the patient groups (Supplementary Figs. 9 and 10a). The white blood cell count and lactate level at time point *t*_1_, which are two other established markers of septic shock, did not differ between the patient groups (Supplementary Fig. 10b). We also looked at the APACHE-II score and SOFA score, which are two important clinical metrics for sepsis and septic shock, and found that they also could not be used to reliably stratify the three patient outcomes (Supplementary Fig. 11).

Remarkably, all patients with low GN at *t*_1_ (below ~85 CPN/μl) belonged to group C, who eventually died in the ICU. In addition, we observed that group B patients, the late-recovery group, had higher GN levels than those of group C patients at both *t*_1_ and *t*_2_ (Fig. 5b; Supplementary Fig. 9). This suggests that dynamic measurements of GN at finer temporal resolution may provide insight into the underlying pathophysiology in patients with resolved or ongoing shock.

Given the success of the decision-tree classifier, we tested if patient clinical grouping could be predicted based on our molecular measurements. We used k-fold cross-validation to train a prediction model using data from *t*_1_ only. We found that the average prediction accuracy was greater than 90% (Supplementary Fig. 12). We also compared the decision-tree classifier with two other linear classifiers (linear discriminant analysis and logistic regression) and found the decision-tree classifier to be overall more accurate, and its variation in accuracy between validation sets is smaller. This is probably because of the relatively small sample size in our study: by setting the number of layers for the decision tree classifier, it is less prone to overfitting than the other two linear classifiers. We note that the initial patient classification was based on clinical characteristics obtained by independent clinicians that did not have access to the molecular measurements of our dPLA/dPCR pipeline, eliminating the possibility of classification-related confounders in these groups. Further, the decision-tree analysis only used the measurements from *t*_1_, when the patients’ clinical courses (eventual recovery or death) were unknown at the time of sample collection. However, further studies with a larger cohort of patients are needed to definitively conclude if the biomarker profiles we found are generally applicable.

### Importance of time-dependent measurements in septic shock

IL-6 is an important proinflammatory cytokine involved in sepsis. Although a previous study found some correlations between IL-6 level and septic shock patient mortality^37^, IL-6 has not been useful for predicting individual patient outcomes^12^. Here, we propose that these two observations can be reconciled: we found that the dynamic changes in IL-6, and not its static absolute level at a given time point, were strongly correlated with individual patient mortality (Fig. 5c–e). Our time-dependent digital measurements showed that patients who died (group C) showed an increase in IL-6 between time points *t*_1_ and *t*_2_, while all patients who recovered (group B) showed a strong decrease in IL-6. The IL-6 level at time point *t*_1_, on the other hand, was not correlated with patient survival or death.

We also found that the temporal change in G-CSF levels was slightly correlated with the patient mortality (*P* value = 0.07) (Supplementary Fig. 13). Temporal changes in procalcitonin, IL-1β, and IL-8 were not correlated with patient mortality.

Our observations highlight the potential importance of dynamic measurements (i.e., longitudinal monitoring) in classification of septic shock outcomes (Fig. 5c). Sensitive quantification of the early changes in IL-6 levels may allow anticipation of patient mortality at a much earlier time point. Our dPLA/dPCR protocol was able to detect differences in IL-6 levels as small as 0.04 pg/ml, showing the potential suitability of our method for early diagnosis, monitoring, and treatment of this deadly disease.

## Discussion

Here, we present the development of new digital molecular assays for sensitive and multiplexed quantification of proteins (IL-6 and TNF-α) and nucleic acid targets (GN, GP, and *bla*_TEM_ genes) in patients. We emphasize the potential of our approach in characterizing human biofluid samples and serving as an effective longitudinal analytical method, and how their measurements can reveal observations and potential biomarkers that warrant further investigation. We were able to quantify host cytokines using as little as 1–10 μl of human BALF and plasma samples with femtomolar sensitivity. The increased sensitivity allowed us to monitor septic shock patients, and to detect a small amount of cytokines in highly diluted BALF samples from patients with asthma. We demonstrated how our assays could be utilized to detect dynamic markers of infectious pathogens and host inflammatory response, and how this information could be used to enable accurate prediction of septic shock patient outcomes.

Our study improved the sensitivity, dynamic range, and multiplexing capabilities of digital protein measurements using dPLA (Supplementary Table 1). The sensitivity we achieved is better than many commercially available ELISA kits (Supplementary Tables 3 and 4). We developed two specific assays for the cytokines IL-6 and TNF-α, as they are key molecules reflecting the level of inflammatory response during infections^14,15^. After an infection, TNF-α and IL-6 protein levels in tissues and blood increase rapidly^15^. TNF-α triggers the expression of chemokines for the recruitment of neutrophils to the infection site^38^. IL-6 is not only a proinflammatory cytokine but also an important biomarker for the evaluation of cancer, acute cellular rejection, pneumonia, asthma, and bacteremia^39,40^. The method we present here thereby provides a valuable tool set for monitoring patient responses in a wide range of diseases.

Compared with conventional microbiological detection methods, our droplet digital-PCR-based assay can detect bacteria that are difficult to culture. Our assay for measuring cytokines represents an important step toward an effective technology for longitudinal monitoring of host responses: the assay has a high resolution (we were able to detect a difference of 0.04 pg/ml in IL-6 levels) and is compatible with a small sample volume (we could use as little as 1 μl per measurement). While assays for IL-6 and bacterial DNA can be performed in 2 h, the TNF-α dPLA assay currently requires 7 h to obtain reliable results due to an antibody incubation step. We used overnight incubation to maximize TNF-α signal in this study. We anticipate that the incubation step may be shortened by using a different clone of TNF-α antibody.

Antimicrobial resistance is a growing public health threat. The broad-spectrum β-lactamases are the most abundant plasmid-mediated antibiotic-resistance enzymes^41^, and *bla*_TEM_ is a major β-lactamase gene. Here, we optimized a triplex droplet digital-PCR (ddPCR) assay that can discriminate as small as 1 *bla*_TEM_ gene copy number difference in a microliter of sample, and used it to detect the emergence of antibiotic resistance in various human samples. Our assay produces a result within 2 h, which is much faster than conventional culture-based techniques that take several days.

To demonstrate the potential of our approach in guiding the clinical care of patients, we first collected BALF samples from patients with asthma, and found that these samples are enriched in GN bacteria and IL-6 compared with healthy control individuals. These results are in agreement with previous studies, which showed that a higher GN load could be due to a difference in the airway microbiome of some asthmatic subjects, particularly those with lower levels of type-2 inflammation^42–44^, and that IL-6 levels may reflect the proinflammatory state of the lung^34^. Measuring the load of GN bacteria and the levels of IL-6 may allow further refinement of the asthma diagnosis as type 2 versus non-type 2, for exploration of other asthma phenotypes (e.g., the IL-6-high phenotype recently described)^40,45^, and may also inform which patients could be treated more aggressively for ongoing asthmatic inflammation.

Early detection and the accurate use of antibiotics are paramount to treating sepsis and septic shock^46^. Our technology enables early detection of infection by quantifying bacterial load in plasma, and allows monitoring of the patient inflammatory response by measuring cytokines sensitively. By combining our molecular measurements at the first time point with machine-learning analysis (Fig. 5a; Supplementary Fig. 12), we were able to classify patients with septic shock into three clinical groups that reflected their outcomes (early resolution, late resolution, and death), with over 90% accuracy, thereby demonstrating the potential power of our approach in managing this devastating disease.

The combination of characterization of pathogens and dysregulated host response in an assay carries significant diagnostic and prognostic advantages. This is in contrast to the current approach that relies primarily on clinical markers of disease. Biomarker use has been studied extensively, but has yielded disappointing results. No biomarker has been shown to be accurate enough to diagnose or predict prognosis^47,48^. This is reflected in the Sepsis-3 guidelines, which do not recommend the use of any biomarker for diagnostic or prognostic purposes. In these guidelines, the use of procalcitonin is weakly recommended only to guide antibiotic discontinuation, but more recently even this has not been shown to improve outcomes and is not recommended in the management of common infections, such as pneumonia^49–51^.

A meta-analysis study suggested that an increase in IL-6 levels could be associated with septic patient prognosis^52^. Here, we show that the changes in IL-6 plasma levels indeed have a strong correlation with patient mortality and survival. The differences in IL-6 levels between patient outcomes became clear only at time point *t*_2_ (Fig. 5), highlighting the importance of temporally resolved, longitudinal measurements. Although time point *t*_2_ occurred 24–48 h after ICU admission in this study, we plan to explore collecting additional samples at earlier time points in future studies.

Our decision-tree analysis of GN and TNF-α levels at *t*_1_ classified 32 patients into three clinical groups with high accuracy (Fig. 5a). Interestingly, we found a counterintuitive relationship between GN levels and patient outcome: patients who eventually died had lower GN levels at time point *t*_1_ than those who survived. This would support the idea that mortality in sepsis is mainly due to inflammatory cytokine response rather than by the direct effects of the pathogens. This idea is further supported by increasing levels of IL-6 in the patients who died. While the bacterial DNA measurements do not indicate the source and location of the bacterial entry, which can include the endogenous, nonpathogenic GN microbiota in the patient samples, our analysis shows that septic shock patient outcome can still be effectively predicted in this cohort. Current commercial platforms for bacteria identification, such as the LiDia system from DNAe, only provide identity information of the bacteria and not their abundance, the latter of which we found to be predictive of septic shock patient outcomes.

The correlation between increasing IL-6 levels, low GN bacteria levels, and patient death signifies an excessively strong proinflammatory response by the patients. This suggests, at least for the patients involved in this study, that the cause of death in patients with septic shock may be the excessive cytokine response rather than the direct effects of the bacteria on the host^52^. Further studies with a larger cohort are needed to determine whether this is generally true for all patients with septic shock, and whether these markers could be used to diagnose sepsis at an earlier stage.

In summary, we presented a method for identification of both microbes and cytokines from extremely small volumes of biofluids with unprecedented sensitivity. As proof of concept, we have used this method to develop a decision-tree model that accurately predicts outcomes among hospitalized patients. We anticipate that this approach will illuminate new biologic insights and allow personalized therapeutic approaches in the care of patients with infections.

## Methods

### Study design

We developed highly sensitive and specific dPLA/ddPCR assays for monitoring host (IL-6 and TNF-α) and pathogenic biomarkers (GN/GP 16S rRNA genes, and *bla*_TEM_ gene), respectively. We used two cohorts: the first cohort had 23 patients with asthma, 11 healthy individuals, from whom BALF samples were collected. The second cohort had 32 septic shock patients admitted to the ICU. Healthy individuals to establish the background levels of circulating bacterial DNA and cytokines were enrolled from among volunteers. Septic shock patients were divided into three phenotype groups: group A patients (*n* = 7) recovered within 48 h, group B patients (*n* = 13) required >48 h to recover, and group C patients (*n* = 12) died in the ICU on vasopressors. Recovery was defined as resolution of shock, no longer requiring vasopressors to maintain a mean arterial pressure greater than 65 mmHg. We analyzed plasma samples that were collected at multiple time points. All DNA (GN, GP, and *bla*_TEM_) and protein measurements (IL-6 and TNF-α) were done in either duplicate or triplicate at ddPCR and dPLA levels, respectively.

### BALF sample collection

BALF sample collection followed a previous prospective from a study that was approved by the Institutional Review Board at the University of Chicago^53^. All subjects provided written and informed consent for participation into the study. The 23 subjects with asthma (average age: 46 [38–53], 53% female, 65% white, 30% black, and 4% others) were recruited in order to provide a wide range of disease states. The subjects met criteria defined by the EPR 3 Guidelines on Asthma^54^. Eleven control subjects were in good health, and they did not use respiratory-related medication. Bronchoscopy was performed on subjects within 4 weeks of their visit as described in previous works^55,56^.

### Plasma sample collection from septic shock patients

The protocol for this study (18-1163) was approved by the University of Chicago Institutional Review Board for human studies. The study was performed in accordance with the ethical standards in the 1964 Declaration of Helsinki and in the later amendments therein. We obtained written consent from the study subjects or from their surrogates. Plasma collection was conducted at the University of Chicago Medical Center. Patients were diagnosed with septic shock based on criteria established by the Sepsis-3 guidelines^2^. Briefly, adult patients >18 years of age admitted to the Medical Intensive Care Unit of the university who required vasoactive medication support for blood pressure and had a suspected infection were approached for enrollment within the first 24 h of diagnosis. In total, 30 mL of blood was collected in sodium citrate tubes (as anticoagulant) at three distinct time points: time point *t*_1_ (at enrollment), time point *t*_2_ (24 h after enrollment), and time point *t*_3_ (upon resolution of septic shock). As part of the study protocol, patients were enrolled within the first 24 h of shock. Given that standard of care at our institution is to administer antibiotics within 3 h of sepsis suspicion, all patients had received antibiotic therapy prior to sample collection.

If patients no longer required vasoactive medications at 24 h after enrollment, no further samples were drawn, and thus patients had only two samples (group A). If patients died never having resolved their septic shock, no third sample was drawn (group C). Thus, all samples drawn at time points *t*_1_ and *t*_2_ are within 24 h of each other, but samples drawn at time point *t*_3_ were drawn at varying times, depending on when the patient ceased requiring vasoactive medications (group B). Within 2 h of collection, samples were centrifuged at ~500 *g* for 15 min to isolate plasma. They were immediately stored at −80 °C. Clinical data were abstracted from the patient’s medical record. Applied Physiology and Chronic Health Evaluation-II (APACHE-II) and Sequential Organ Failure Assessment (SOFA) scores were calculated on the day of enrollment^57–60^. SOFA scores were also calculated on each day of sample collection.

### Reagents

We used the following consumables: Eppendorf 96-Well twin.tec PCR plates (Eppendorf, #951020362), 0.2-μl thin-walled PCR tubes (Thermo Fisher Scientific, #AB-0620), 0.2-μl thin-walled PCR strips (Thermo Fisher Scientific, #AB-1182), and 1.5-ml microcentrifuge tubes (Ambion, #AM12450).

The biotinylated antibodies (BAB), recombinant protein standards were from R&D Systems: biotinylated anti-human IL-6 polyclonal goat antibody (#BAF206), biotinylated anti-human TNF-α polyclonal goat antibody (#BAF210), recombinant human (RH) IL-6 (#206-IL-010), RH TNF-α (#210-TA-020), and RH IL-10 (#217-IL-005). Chicken plasma was purchased from Sigma (#G2282236).

### Preparation of proximity probes

Proximity probes were prepared according to the protocol of TaqMan Protein Assays Open Kit (Thermo Fisher Scientific, #4453745). 2 μl of 1 mg/ml BAB stock was diluted to a concentration of 200 nM by mixing with 60.5 μl of antibody dilution buffer (ADB) (Thermo Fisher Scientific, #4448571). 5 μl of 5′ and 3′ prox-oligos (200 nM each) were separately combined with 5 μl of 200 nM of BAB, and incubated at room temperature (RT) for 1 h to make 10 μl of 100 nM 5′ proximity probe A and 10 μl of 100 nM 3′ proximity probe B, respectively. Each probe was then diluted to 10 nM by mixing with 90 μl of assay probe storage buffer (brought up to room temperature before mixing), incubated at RT for 20 min, and kept at −20 °C.

### dPLA protocol

All dPLA reagents were parts of the TaqMan Protein Assays Open Kit unless otherwise stated. First, we prepared the protein solution by diluting the sample five-fold in the sample dilution buffer (SDB, see below for more details), and prepared the assay probe solution (APS) by combining 1 μl of proximity probe A, 1 μl of proximity probe B, and 23 μl of assay probe dilution buffer.

Next, we combined 2 μl of protein solution with 2 μl of APS (200 pM/probe), and incubated the mixture at 37 °C for 1 h (for TNF-α, the mixture was overnight incubated at 4 °C). After probe incubation, we prepared the ligation solution by combining with 50 μl of 20× ligation reaction buffer with 909 μl of nuclease-free water, and 1 μl of DNA ligase (1×, in ligase dilution buffer). Then, 96 μl of ligation solution was added to 4 μl of the protein/probe solution; the mixture was incubated at 37 °C for 10 min. To stop ligation, we either heated the solution at 95 °C for 5 min for IL-6 dPLA, or performed protease digestion for TNF-α. The protease digestion was performed by adding 2 μl of 1× protease prediluted in PBS, incubated at 37 °C for 10 min and 95 °C for 15 min. In total, 20 μl of ddPCR reaction mixture was prepared by combining 9 μl of the final PLA solution with 10 μl of 2× ddPCR Supermix (Bio-Rad, #186-4033 or #186-3023, the latter was required for multiplex digital assay) and 1 μl of 20× Universal PCR Assay solution. The mixture was pipette-mixed and emulsified according to the manufacturer’s instructions (Bio-Rad, #1864002). The droplets were sealed and thermally cycled as the following: 95 °C for 10 min; 40 cycles of 94 °C for 30 s and 60 °C for 1 min; 98 °C for 10 min (ramping speed was 2.5 °C/s). Finally, the positive droplets were counted by a droplet reader (Bio-Rad, #1864003), and the analyte concentration was estimated by QuantaSoft Analysis Pro software (Bio-Rad, v.1.0.596).

Because the software only returns the lower and upper limits of the 95% confidence interval (CI), we estimate the standard deviation (SD) by using the following formula:

1 $$SD = \frac{{CI_{{\mathrm{max}}} - CI_{{\mathrm{min}}}}}{{2 \ast 1.96}} \times \sqrt n$$

where *CI*_max_ and *CI*_min_ denote the upper and lower limits of the 95% confidence interval (given as TotalConfMax and TotalConfMin by the software), respectively, and *n* is the number of replicates.

### Preparation of dPLA sample dilution buffer

For IL-6 assay in plasma samples, we used SDB-II (Thermo Fisher Scientific, #4483013). For TNF-α assay in plasma samples, we prepared a homemade buffer (HMB) based on Nong’s buffer^29^: 1× PBS, 1 mM d-biotin (Sigma, #47868), 0.1% gelatin (Aurion, #900.03) 0.05% vol/vol Tween-20 (Sigma, #P9416), 100 nM goat IgG (Thermo Fisher, #50643345), 0.1 mg/ml salmon sperm DNA (Invitrogen, #15632-011), and 4 mM EDTA (Invitrogen, #AM9261). For both IL-6 and TNF-α quantification in BALF samples, we used TM buffer (Biochain, #K3011010-1) diluted to 0.1× in Milli-Q water, and enriched with 0.5% gelatin as the sample dilution buffer (SDB).

### DNA extraction from human samples

DNA was extracted from all BALF and plasma samples with QIAamp cador Pathogen Mini Kit (Qiagen, #54104) according to the manufacturer’s instructions. First, 100 μl of sample was combined with 100 μl of PBS and 20 μl of proteinase K. Next, 100 μl of buffer VXL was added, and the mixture was incubated at RT for 15 min. Then, it was combined with 350 μl of buffer ACB and mixed thoroughly, and the lysate was loaded to the QIAamp Mini column. The column was centrifuged at 6000 *g* for 1 min, washed with AW1 and AW2 buffers, and centrifuged after each wash (6000 *g*, 1 min) to remove the filtrate. At the end, we added 100 μl of AVE, incubated for 1 min, and spun at 20,000 *g* for 1 min. This final eluate was used as the analyte solution. In addition, we measured the background signal of the DNA extraction kit (to check for contaminations) by performing DNA extraction using DNA-/RNA-free PBS. The average background signal was then subtracted from all human samples’ measurements.

### Preparation of DNA samples for optimization of ddPCR

To prepare the standard for *bla*_TEM_, we inserted the pBR322 TEM-1 plasmid (a gift from Toprak’s Lab) to a strain of competent *E. coli* (New England Biolabs, #C2987H). Plasmid-positive *E. coli* colonies were used as *bla*_TEM_-positive control. After overnight growth, the bacteria were harvested, and their plasmids were isolated using QIAprep Miniprep (Qiagen, #27104). The plasmid concentrations were measured with a Qubit Fluorometer (Invitrogen, #1.0).

For the validation experiments of the specificity of the ddPCR primers and probes for GN and GP quantification, we used *E. coli* (NEB, #C29871) and methicillin-resistant *S. aureus* (ATCC, #BAA-1756) as the Gram-negative and Gram-positive samples, respectively. The bacteria were grown in a shaking incubator until they reached an optical density of OD_600_ = 1.2. Next, the bacteria were pelleted at 10,000 rpm at 4 °C for 10 min, resuspended in sterile PBS, and centrifuged at 10,000 rpm at 4 °C for 10 min. Then, the bacteria were resuspended in sterile water, and boiled for 5 min. Finally, the samples were centrifuged at 12,000 rpm for 10 min, and the supernatants were stored at −20 °C.

For the experiments on the relationship between ddPCR quantification and the number of colonies forming units per microliter, we used the same *E. coli* and *S. aureus* strains. After overnight growth, the bacteria were diluted with PBS such that the optical density value OD_600 _= 0.25, and split into two samples, one for measuring the CFU of the bacteria, and one for DNA extraction. To count the CFU value, the first sample was tenfold serially diluted nine times, and 50 μL of each of the last five dilutions were plated in duplicate on tryptic soy agar plate for overnight incubation at 37 °C, followed by counting in the following morning. The bacteria’s genomic DNA was extracted using the DNeasy Blood & Tissue Kit (Qiagen, #69504), and the extracted DNA was kept at −20 °C for qPCR/ddPCR experiments.

To prepare the DNA standards for calibration curves, the gBlock fragments of interest (GN, GP 16S rRNA genes, and *bla*_TEM_ gene, manufactured by IDT) (Supplementary Table 5) were diluted in AVE buffer with 0.1 mg/ml salmon sperm DNA.

### ddPCR protocol

DNA could be quantified using either EvaGreen or TaqMan probe by ddPCR. Each 20-μl ddPCR EvaGreen reaction mixture contained 1× ddPCR EvaGreen supermix (Bio-Rad, #186-4033), 150 nM of each primer, and samples. Each 20-μl ddPCR TaqMan probe reaction mixture contained 1× ddPCR Supermix for Probes (Bio-Rad, #186-3023), 900 nM of each primer, 200 nM of the appropriate TaqMan probes (see Supplementary Table S1), and sample. The mixture was emulsified according to the manufacturer’s instructions, and the following thermal cycling program was used for GN/GP quantification: 95 °C for 10 min; 40 cycles of 94 °C for 30 s and 62 °C for 1 min; 98 °C for 10 min (ramping speed was 2.5 °C/s). For *bla*_TEM_ quantification, the thermal cycling program was 95 °C for 10 min, 40 cycles of 94 °C for 30 s, 50 °C for 15 s, and 70 °C for 20 s; 98 °C for 10 min (ramping speed was 2.5 °C/s). Then, the droplets were analyzed and quantified (see “dPLA” section for details). The sequences of the primers/probes are given in Supplementary Table 5.

### Simultaneous quantification of GN and GP bacteria with ddPCR

Each 20-μl ddPCR reaction mixture contained 1× ddPCR Supermix for Probes, 900 nM of each universal primer (i.e., Bac_fwd and Bac_rev), 200 nM of HEX-labeled GN probe (GN_probe), 200 nM of FAM-labeled GP probe (GP_probe), and the sample. Following emulsification, we used the same thermal cycling program as the simplex ddPCR GN/GP assay above. Then, the droplets were analyzed by the reader (see dPLA section for details).

### Simultaneous quantification of GN, GP, and *bla*_TEM_ genes

Each 20-μl ddPCR reaction mixture comprised 1× ddPCR Supermix for Probes, 900 nM of each primer (Bac_fwd, Bac_rev, TEM_fwd, and TEM_rev), 340 nM of HEX-labeled GN probe, 180 nM of FAM-labeled GP probe, 380 nM of FAM-labeled *bla*_TEM_ probe (TEM_probe), and the sample. After emulsification, the droplets were heated with the same thermal cycling program as the simplex ddPCR GN/GP assay. In data analysis, the 2D amplitude plot clearly showed four clusters: HEX-positive (GN signal), FAM-medium (GP signal), FAM-high (*bla*_TEM_ signal), and no fluorescence (empty droplets). Each cluster was quantified by automatically/manually setting the appropriate fluorescence amplitude thresholds. The concentration of the analytes was calculated by the software (see dPLA section).

### Simultaneous quantification of IL-6 and DNA targets

This triplex assay only applied to BALF samples was made possible by combining ligation and ddPCR amplification into one single step. The PLA reagents for the triplex assay were from the TaqMan Protein Assay-II kit (Thermo Fisher Scientific, #4483013). First, 2 μl of BALF samples (prediluted in 0.1× TM buffer if necessary) was combined with 2 μl of assay probe solution (proximity probes A and B at 160 pM/probe). It was incubated at 37 °C for 1 h. Next, 2 μl of the mixture was combined with 20 μl of ligation/ddPCR reaction mixture containing 1× Universal PCR Assay-II, 1× Fast master mix-II, 1× Ligation Additive-A, 1× Ligation Additive-B, 1× DNA ligase-II, 900 nM of each bacteria primer (i.e., Bac_rev and Bac_fwd), 150 nM of HEX-labeled GN_probe, and 150 nM of FAM-labeled GP_probe. In all, 20 μl of the mixture was emulsified and heated with the following thermal cycling program: 25 °C for 20 min (ligation reaction); 95 °C for 5 min; 40 cycles of 94 °C for 30 s and 60 °C for 1 min; 98 °C for 10 min (ramping speed was 2.5 °C/s). Finally, the droplets were analyzed by the software, and different clusters were clearly plotted on the 2D amplitude plot (Fig. 2a).

### Multiplexed Luminex assay

The multiplex Luminex kit was custom-designed and sold by R&D Systems (https://www.rndsystems.com/products/luminex-assays-and-high-performance-assays). Standards for calibration were prepared according to the manufacturer’s recommendation. For this kit, standard samples are provided in lyophilized form and reconstituted on the day of the assay, followed by serial dilution to allow creation of a standard curve. Samples were assayed on a 96-well plate, and separate standard curves were generated for each plate. Each patient sample was measured in duplicate.

### Quantification of bacterial DNA targets and host cytokines

Because ddPCR is an absolute quantification technique, we used the ddPCR signal directly as the concentrations of GN, GP, and *bla*_TEM_. Any sample with signal below the LOD as defined below was considered to be 0. For IL-6 and TNF-α, we constructed a linear regression line based on the standards, and converted ddPCR signal to protein concentrations. All concentrations reported in the study were the concentrations in the samples.

### Calculation of LOD

LOD refers to the lowest ddPCR signal that can be distinguished from background. LOD is calculated by adding two times the standard deviation of background signal to either the background signal or the intercept of the linear regression curve, whichever is higher. The background signal in this context is equal to the ddPCR signal that the dPLA or ddPCR assay produces when there is no analyte. All measurements below the LOD were treated as 0.

### Estimation of the assay resolution of IL-6 dPLA

For two IL-6 measurements to be considered distinguishable, the absolute difference between the two measurements must be higher than both two times the standard deviation of the first measurement, and two times the standard deviation of the second measurement. We found that in two patients, one in group A and one in group C, a difference of ~0.04 pg/ml satisfied the above criteria. We also found that this resolution is patient-dependent: some patients had larger differences in IL-6 levels, yet their differences were not considered distinguishable.

### Decision-tree analysis

We used the Python package SciPy package scikit-learn for decision-tree analysis^61^. We first standardized the measurements, and trained the decision-tree algorithm DecisionTreeClassifier with max_depth=2 and the defaults for the other parameters. For k-fold cross-validation, we first separated the data into training and test sets using StratifiedKFold with n_splits=5, standardized the training data set, trained the decision-tree classifier (with max_depth=2), and then classified the test data set that had been standardized using the parameters from the training data set standardization.

### Statistical analysis

All statistical analyses were performed with Python and R software. All statistical tests were done on the raw, nontransformed concentrations.

Because of the zero inflation in the measurements of BALF samples, we made use of a two-part statistical test^62,63^ to evaluate the statistical difference between healthy control subjects and patients with asthma. We wrote an R script (twopart_statistics.R, Supplementary Software 1) to perform the two-part statistical test. A two-part statistical test of two samples tests the composite null hypothesis that the two samples have equal proportions of nonzero values, and the distributions of the nonzero values are equal. To calculate the *P* value for the test, we first calculated the test statistics X = Z^2^ + W^2^, where Z was the continuity-corrected two-proportion Z-test statistics of the nonzero values of the two samples (or equivalently, the zero values), and W was the continuity- and tie-corrected Mann–Whitney *U*-test statistics of the nonzero values of the two samples. (While a *t-* test statistic can be used to calculate W instead, the Mann–Whitney *U* test is more appropriate for skewed data and small sample size.) Then, we calculated the *P* value of X, which follows a Chi-squared distribution of two degrees of freedom.

### Reporting summary

Further information on research design is available in the Nature Research Reporting Summary linked to this article.

## Supplementary information

Supplementary Information

Description of Additional Supplementary Files

Supplementary Software 1

Reporting Summary

## Data Availability

Raw patient data used to generate all main and supplementary figures are available upon request from S.T. The ddPCR, qPCR raw data related to optimization experiements are available on figshare with DOI as 10.6084/m9.figshare.12026871.

## Code availability

The R code used to perform the two-sided two-part statistics test is uploaded as Supplementary Software 1.
