# Supplementary material for: Ultrasensitive digital quantification of cytokines and bacteria predicts septic shock outcomes
Source: Nat Commun. 2020 May 25;11:2607. doi: 10.1038/s41467-020-16124-9 (PMC7248118; doi:10.1038/s41467-020-16124-9)
Supplement: Supplementary file 1 — Supplementary Information [file 41467_2020_16124_MOESM1_ESM.pdf]

Supplementary Information

Ultrasensitive digital quantification of cytokines and bacteria predicts septic shock outcomes

Abasıyanık, et al.

SUPPLEMENTARY FIGURES

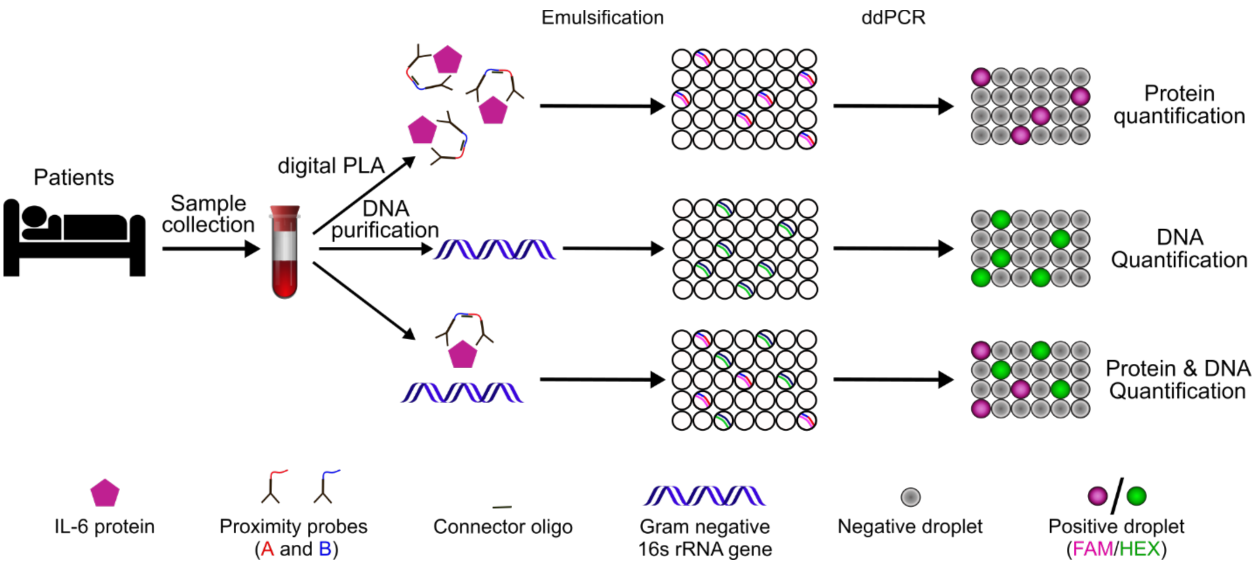

Supplementary Figure 1. Conventional and multiplex dPLA protocols for simultaneous protein and DNA quantification in human samples. For simplex measurements, each collected human sample (BALF or plasma) was split into two, one for nucleic acid quantification by ddPCR and one for protein quantification by dPLA (top and middle flowchart). In dPLA, the protein analyte of interest (IL-6 or TNF-α in our study)[1] were incubated with proximity probes (probes A and B). The proximity oligos were ligated with the help of a connector oligo, resulting in full-length DNA oligos. The generated DNA oligos were partitioned into small water-in-oil droplets, and measured by ddPCR technology. Poisson statistics was then used to estimate the amount of DNA target from the number of positive droplets. For multiplex digital assay (bottom flowchart), we used two different TaqMan probe fluorophores (FAM and HEX). FAM probes were used for the

PLA products and GP 16S rRNA gene, whereas HEX was for GN specific 16S rRNA gene.

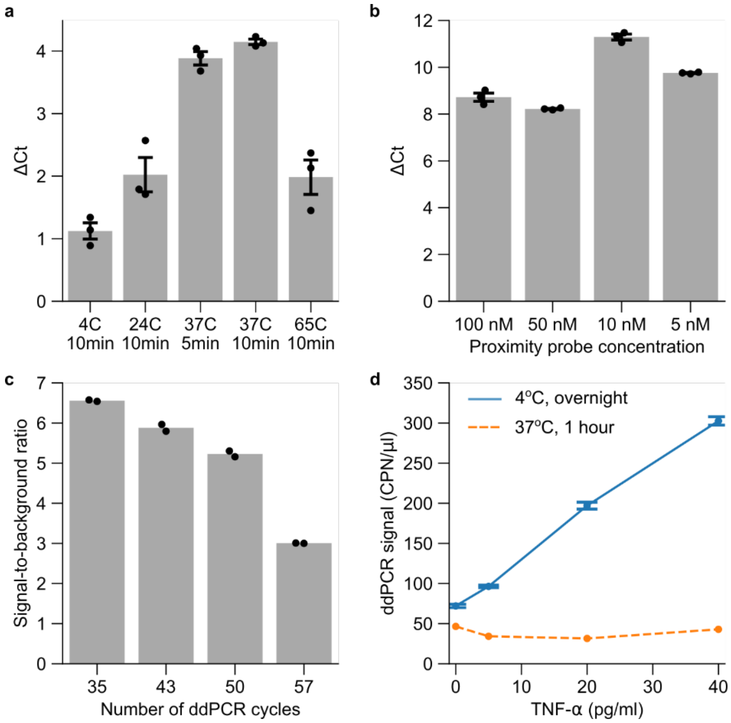

Supplementary Figure 2. Optimization experiments for dPLA Assays. a Effect of ligation conditions in IL-6 PLA. Ligation at 37°C for 10 minutes was found to be the optimal condition. **b** The effect of proximity probe concentration was tested with TNF-α PLA. We found that the combined probe concentration of 10 nM provided the best signal-to-background ratio. In (**a-b**), the PLA products were quantified with qPCR, and the ΔCt value was calculated by subtracting the mean Ct value of the no template control from the mean Ct value of the signal. **c** A TNF-α standard of 0.1 ng/ml was analyzed by dPLA using 4 different ddPCR cycle numbers (35, 43, 50, and 57 cycles). The PCR cycle

number did not affect the signal-to-background ratio, except for 57 PCR cycles. **d** Two different probe incubation conditions were tested for TNF-α dPLA, and overnight incubation at 4°C was found to be necessary and each concentration has 3 technical replicates. (**a, b, d**) Data are presented as mean ± s.e.m. of the measurements. (**c**) Data are presented as mean.

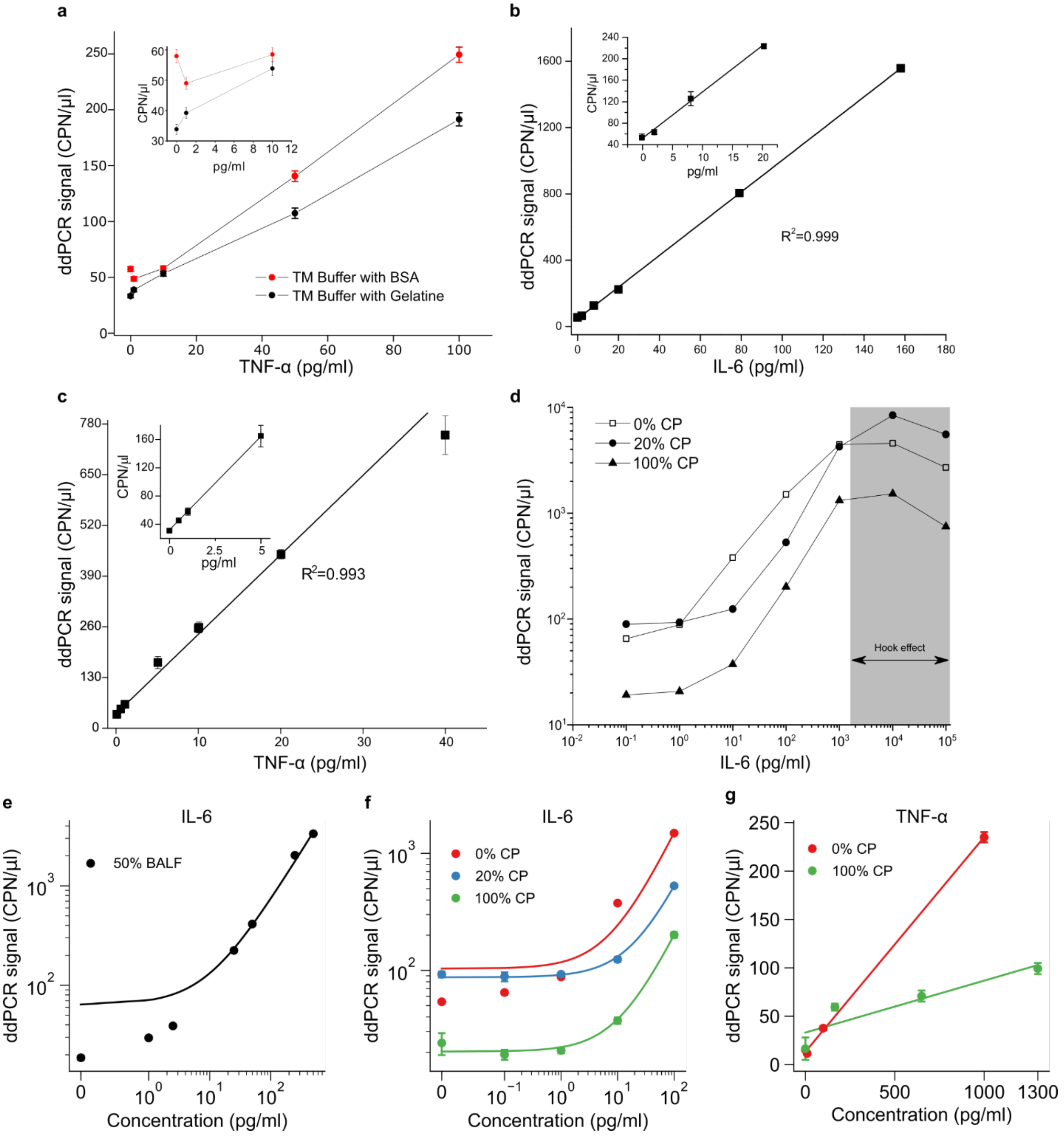

Supplementary Figure 3. Optimization of sample dilution buffers for measuring TNF-α and IL-6 in human samples. a TM buffer was optimized for dPLA assays in a previous study[2]. We enriched that buffer system with gelatin and analyzed TNF-α standards in TM buffers that contain 0.5% gelatin or 0.6% BSA. TNF-α dPLA assay

showed that gelatin enriched TM Buffer produced less noise. **b** Measurement of IL-6 standards diluted in the same buffer as in (a) (TM with 0.6% gelatin) showed high linearity. **c** dPLA quantification of TNF-α standards diluted in Home Made Buffer also showed high linearity. **d** When measuring IL-6 standards diluted in pure Sampled Dilution Buffer-II (i.e., 0% chicken plasma), 20% and 100% chicken plasma (CP), we observed a hook effect at concentration of 1 ng/ml and higher. (Hook effect is the phenomenon in which a higher analyte concentration leads to a lower measurement signal[3].) **e-f** dPLA quantification of IL-6 standards diluted in 50% BALF or various concentration of CP. **g** dPLA quantification of TNF-α standards diluted in 0% or 100% CP. The solid lines in (**e-g**) are the linear regression of the calibration curves. Data are presented as mean ± s.e.m; each concentration has 3 technical replicates.

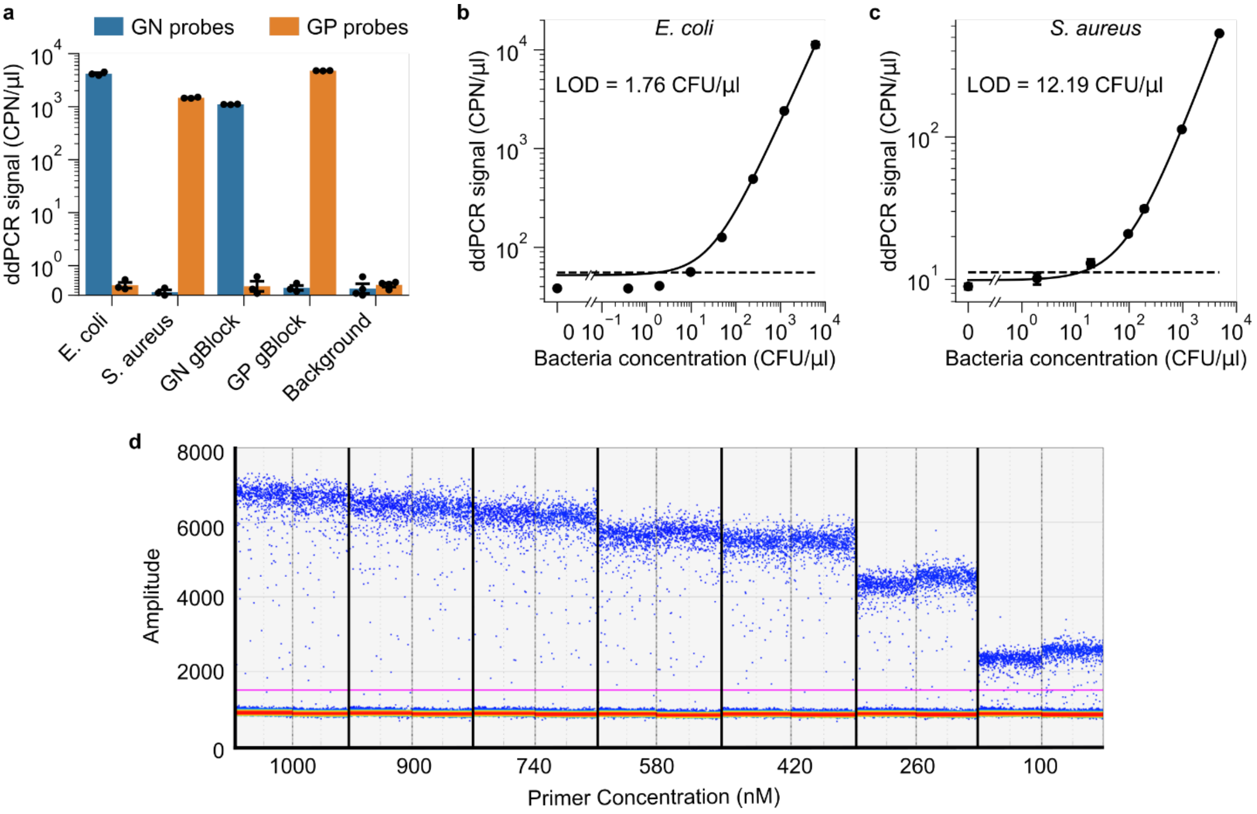

Supplementary Figure 4. Validation and optimization of the universal bacteria primers and gram stain-specific probes. a ddPCR measurements of 4 different sample types (*E. coli, S. aureus*, and GN/GP gBlock standard). In this experiment, each ddPCR reaction mixture contained one of the 4 sample types, the universal bacteria primers, and both GN and GP fluorescent probes (i.e., duplex ddPCR). Note the scale of y axis: it is linear between 0 and 1, and is logarithmic from 1 and higher. **b** and **(c)** An *E. coli* or *S. aureus* culture was used as a standard to check the performance of the assay in term of CFU per 1 µl. **d** ddPCR 1D fluorescence amplitude plot of 55 CPN/µl of GP gBlock standard, using different primers concentrations (1000, 900, 740, 580, 420, 260, and 100 nM combined primer concentration). Data are presented as mean ± s.e.m and each concentration has 3 technical replicates.

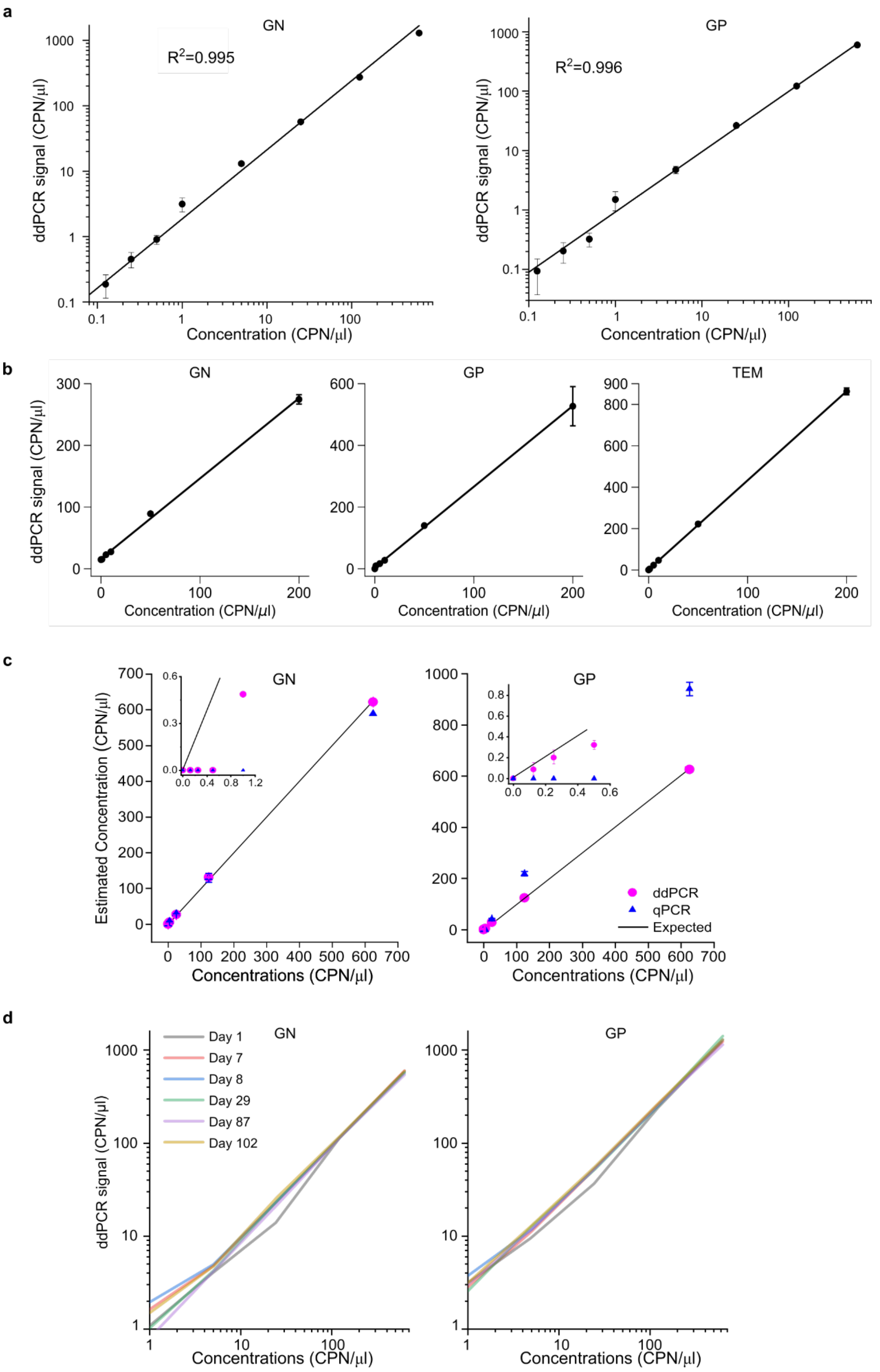

Supplementary Figure 5. Validation of ddPCR for quantification of DNA targets. a Quantification of GN and GP gBlock standards with duplex ddPCR. **b** Quantification of GN, GP and *bla*$_{TEM}$ gBlock standards with triplex ddPCR. **c** Comparison between ddPCR

and qPCR with respect to GN and GP quantification. The plot showed the estimated concentrations of GN and GP gBlock standards, as measured by ddPCR and qPCR, against the actual standard concentrations. The black line showed the expected y=x line. **d** GP and GN gBlock standards were measured with duplex ddPCR at different time points (day 1, 7, 8, 29, 87, and 102) to test the assay's repeatability. Data are presented as mean ± s.e.m and each concentration has 3 technical replicates.

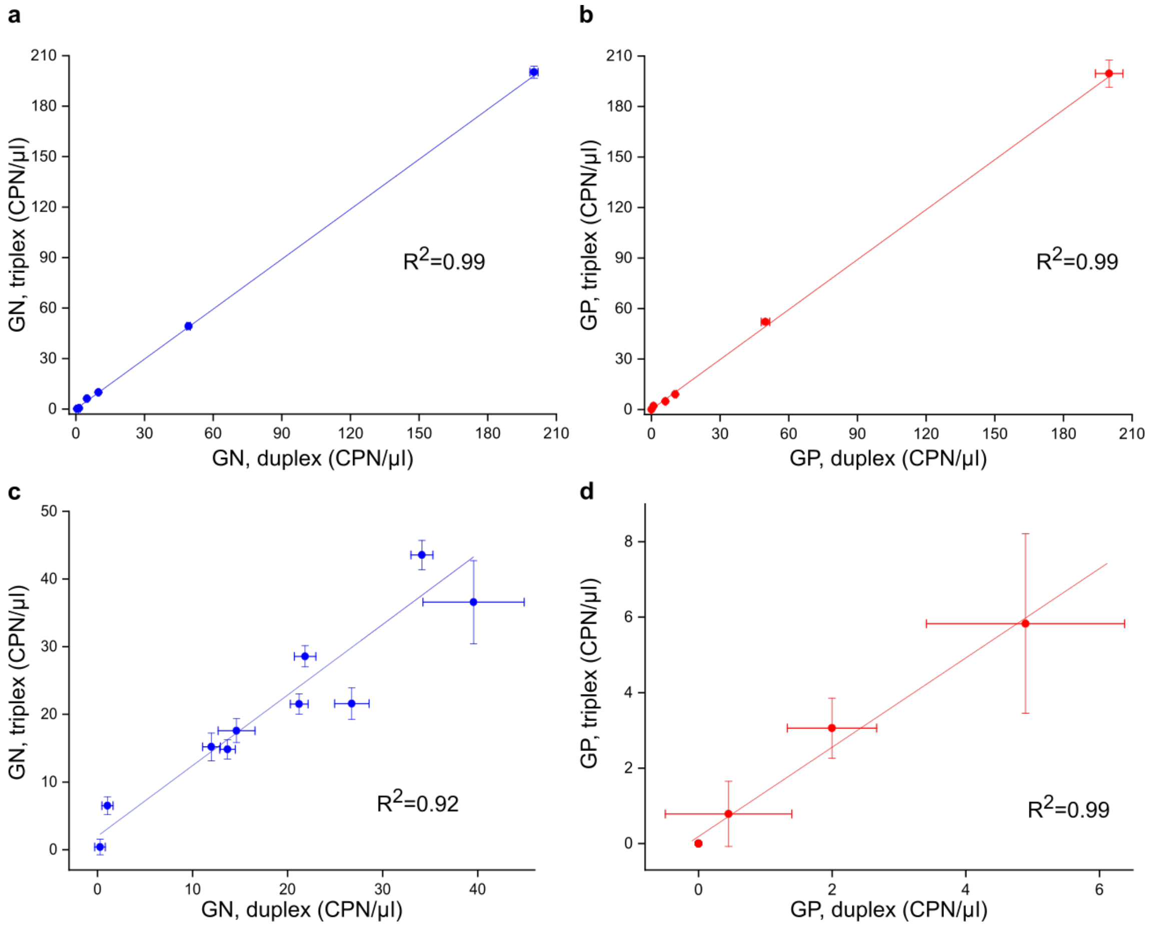

Supplementary Figure 6. Comparison between triplex and duplex ddPCR. a-b Using duplex and triplex ddPCR, we measured GN, GP and *bla*$_{TEM}$ gBlock standards, and 10 BALF samples and estimated their concentration based on standard curve analysis. The

duplex and triplex GP **(a)** and GN **(b)** gBlock standards showed good agreements with each other. In the figures above, X and Y axes demonstrate the estimated concentration of the analyte of interests. **c-d** 10 BALF human samples were analyzed by both duplex and triplex ddPCR to detect **(c)** GN and **(d)** GP bacterial load. These results demonstrated strong agreement between duplex and triplex ddPCR. The shown concentrations in **(a-b)** were calculated in the ddPCR reaction mixture. The bacterial DNA concentrations in **(c-d)** were the concentrations in the human sample. Data are presented as mean ± s.e.m and each concentration has 3 technical replicates.

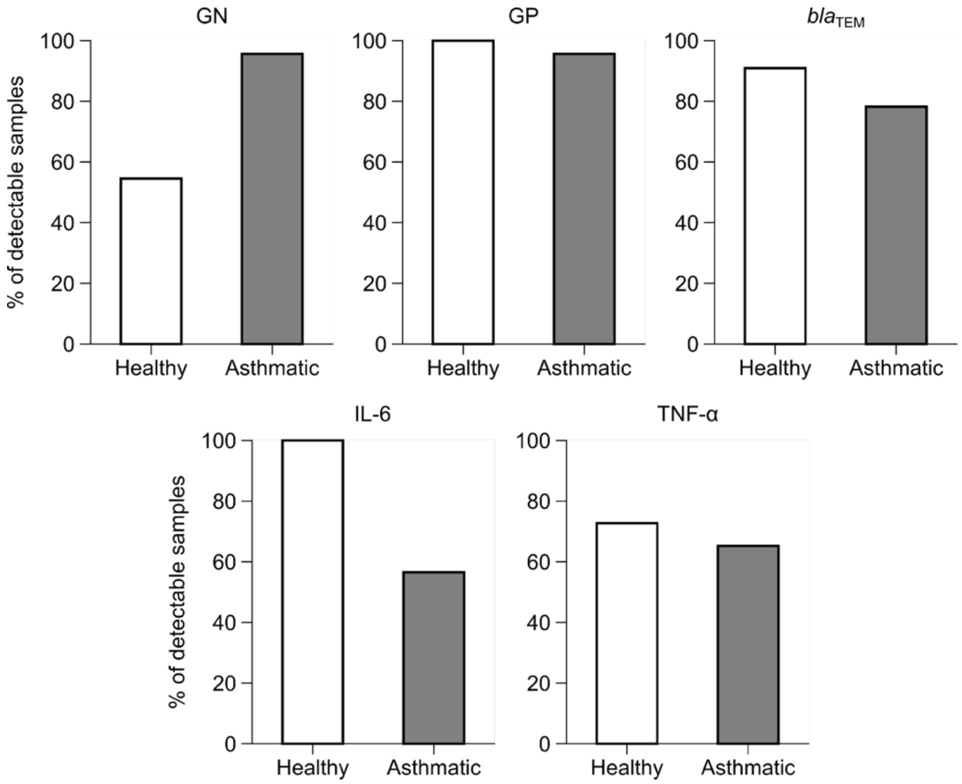

Supplementary Figure 7. Zero-inflation in BALF samples. The percentage of samples whose measurements (GN, GP, bla_{TEM}, IL-6 and TNF-α) were below detectable levels.

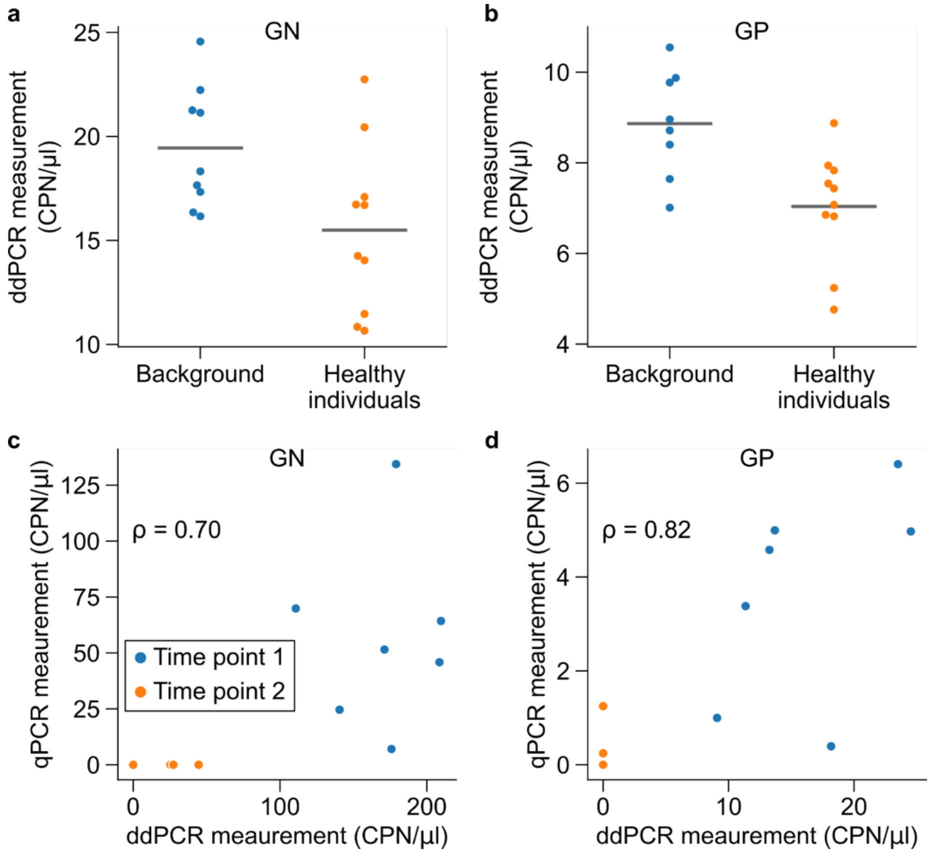

Supplementary Figure 8. Validation of ddPCR assay for GN and GP quantification in human plasma samples. a-b Measured GN and GP levels in healthy human plasma samples (n=10). **c-d** Comparison between qPCR and ddPCR for quantification of GN and GP bacteria in group A (n=7) septic shock patients at time points t_1 and t_2. In **(a-b)**, the grey horizontal lines indicate the average of background signals and healthy individuals' measurements. In **(c-d)**, the Pearson correlation coefficients, ρ, between the two PCR methods for GN and GP quantification were 0.70 and 0.82, respectively.

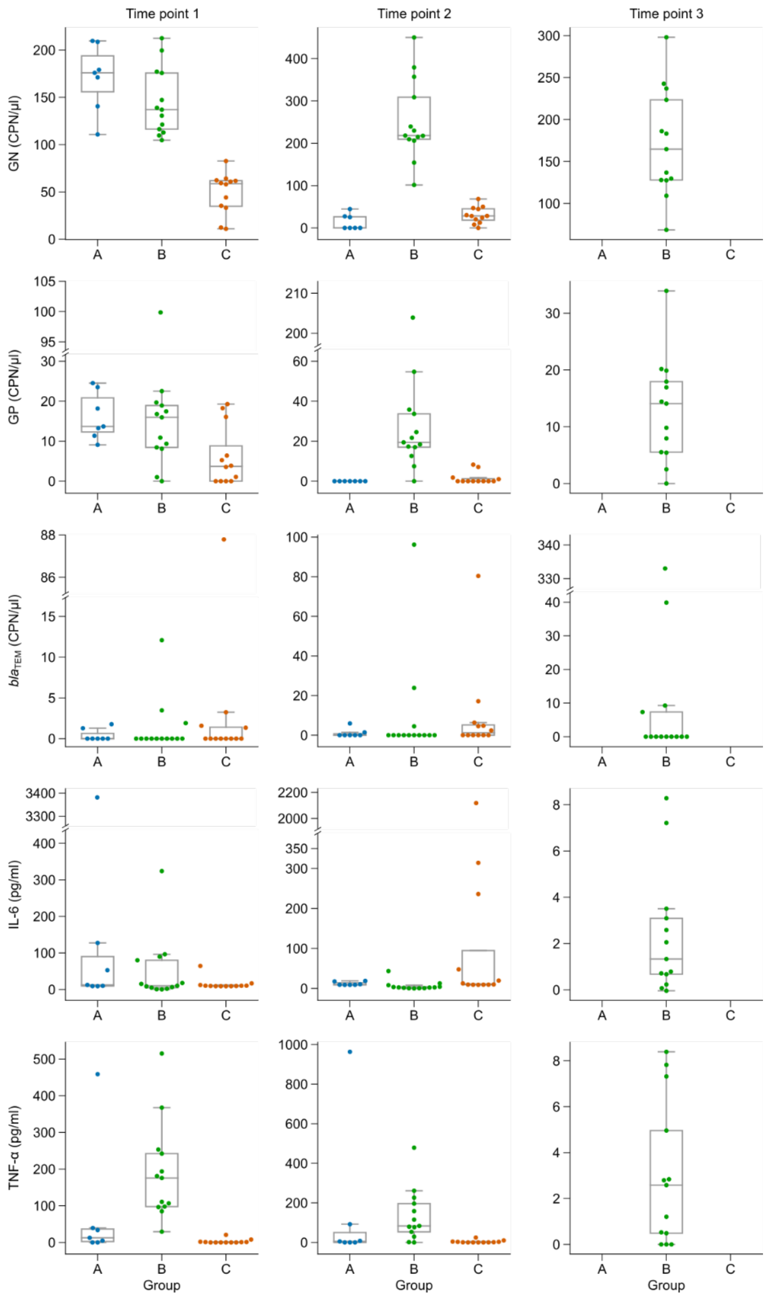

Supplementary Figure 9. Digital measurements of the three septic shock patient groups. Measurements of plasma samples collected at time points t_1, t_2 and t_3 from groups A (n=7), B (n=13) and C (n=12). GN, GP and *bla*$_{TEM}$ levels were measured by ddPCR, and IL-6 and TNF-α levels were measured by dPLA. The box shows the lower

and upper quartile values, the line inside the box shows the median, and the whiskers extend to the value 1.5X the interquartile range beyond the box.

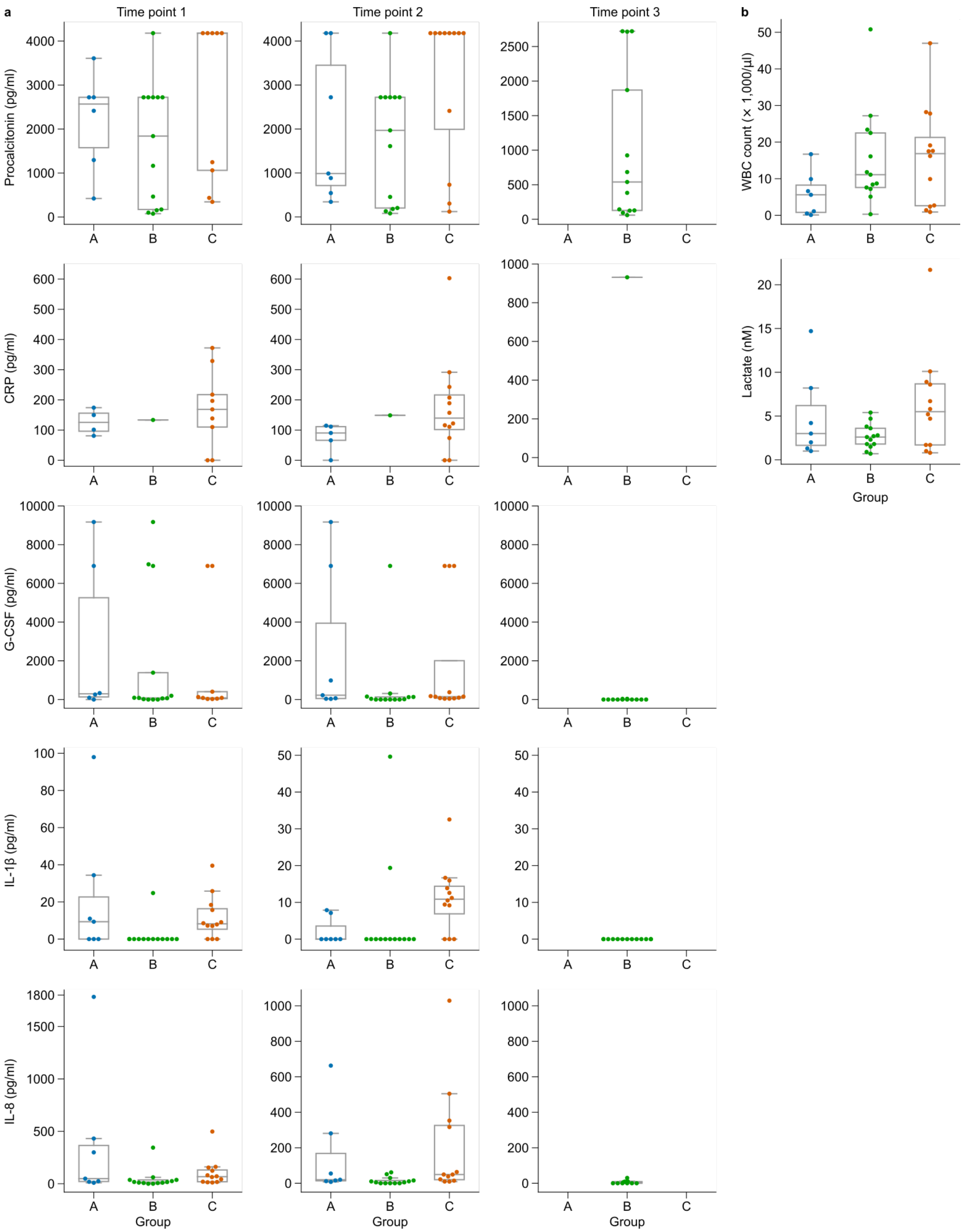

Supplementary Figure 10. The levels of additional biomarkers of the three septic shock patient groups. a The levels of procalcitonin, CRP, G-CSF, IL-1β and IL-8 in plasma samples collected at time points t_1, t_2 and t_3 from groups A (n=7), B (n=13) and C

(n=12). The biomarkers shown in this figure were measured with Luminex bead-based multiplex assay. Note that for group B, the CRP measurement was available from only one patient. **b** The white blood cell (WBC) count and lactate level at time point t_1. The box shows the lower and upper quartile values, the line inside the box shows the median, and the whiskers extend to the value 1.5X the interquartile range beyond the box.

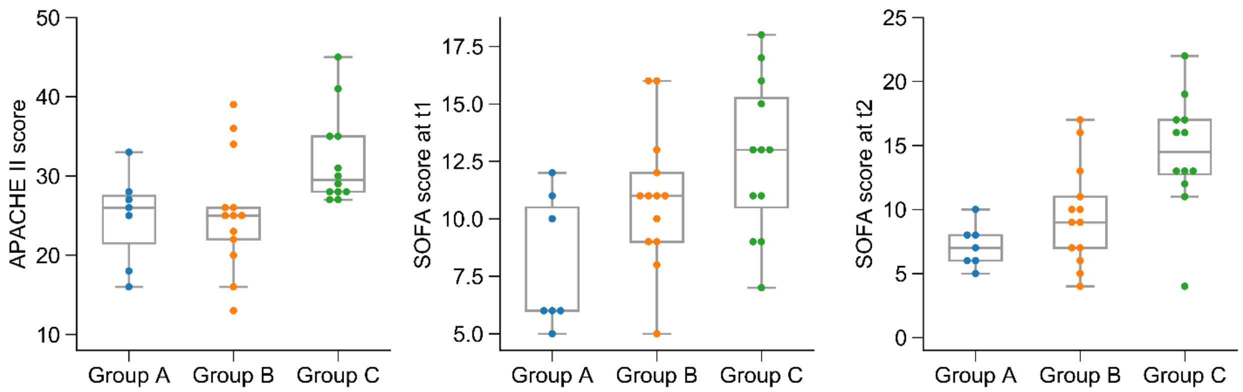

Supplementary Figure 11. The APACHE II scores and the SOFA scores of group C patients (n=12) (the patients who will die) are on average higher than the other two groups (Group A, n=7 and Group B, n=13). However, there is no clear threshold that can be used to distinguish the patient groups. Therefore, the SOFA and APACHE II scores are not as predictive as our digital PLA measurements with regards to septic shock patient outcome. The box shows the lower and upper quartile values, the line inside the box shows the median, and the whiskers extend to the value 1.5X the interquartile range beyond the box.

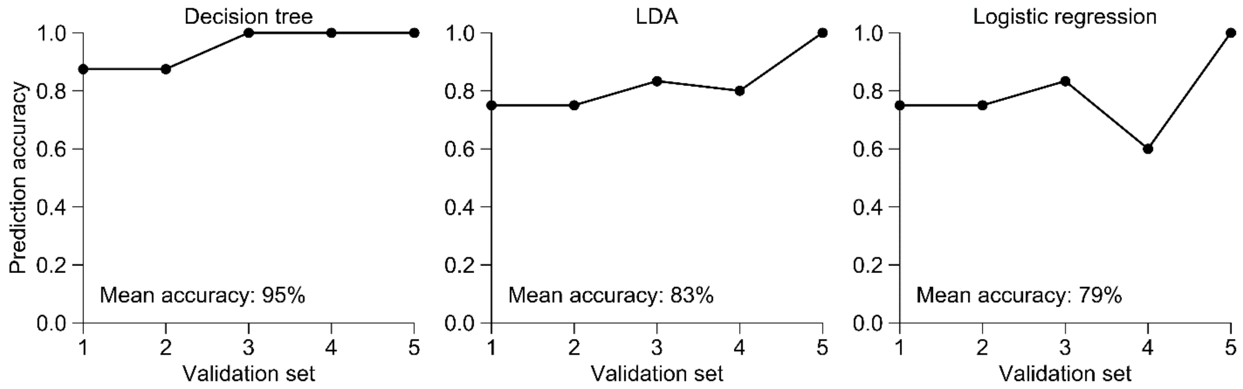

Supplementary Figure 12. k-fold cross-validation of patient group prediction. The patient groups (groups A, B or C) were predicted using time point t_1 measurements. The

accuracy of the decision tree classifier was estimated and compared to that of linear discriminant analysis (LDA) and logistic regression, using k-fold cross-validation (k = 5).

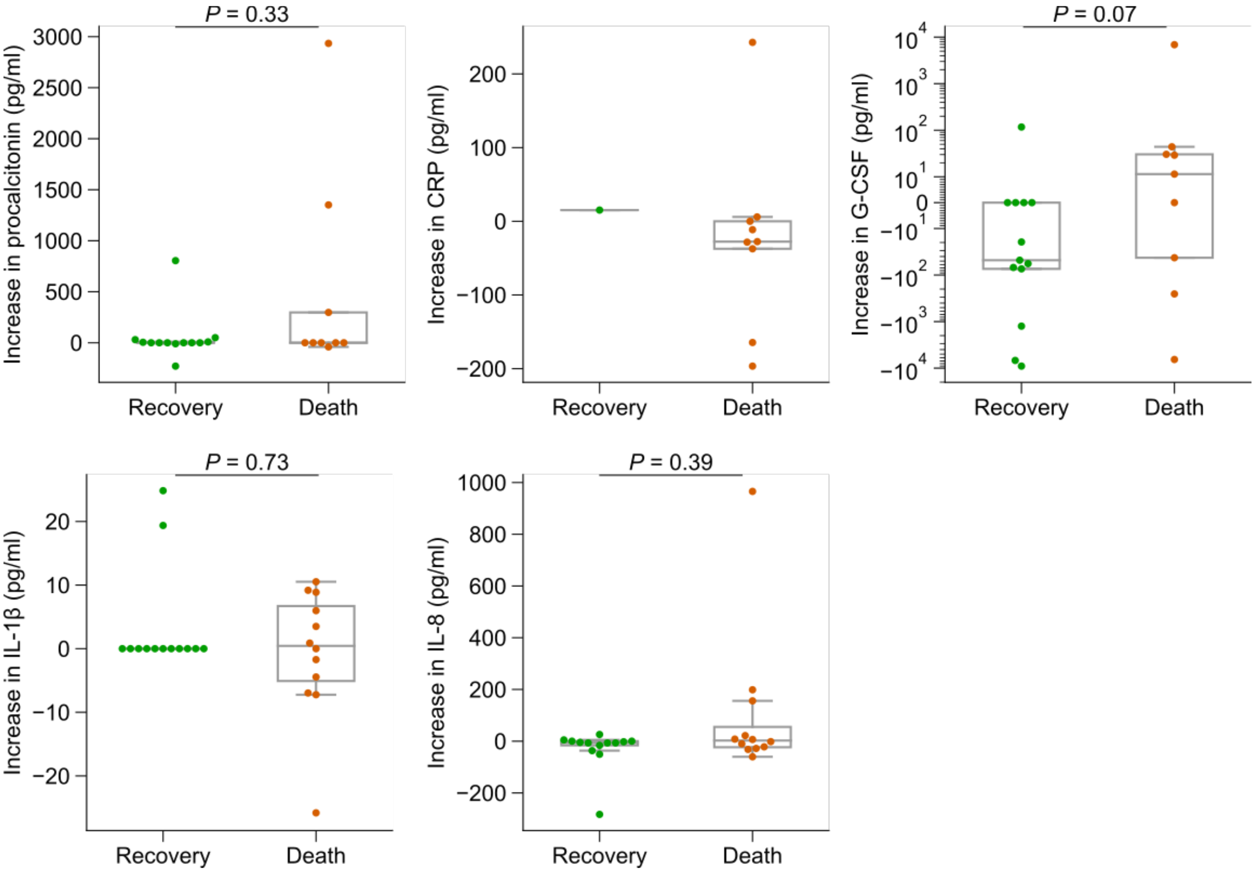

Supplementary Figure 13. Relationship between temporal changes of additional biomarkers and mortality. Increase in the levels of the five additional markers (procalcitonin, CRP, G-CSF, IL-1β and IL-8) between time points t_1 and t_2 of the patient group who recovered (group B, n=13) or died (group C, n=12). Note that for the recovery group (group B), only one t_2 CRP measurement was available. The box shows the lower and upper quartile values, the line inside the box shows the median, and the whiskers

extend to the value 1.5X the interquartile range beyond the box. P value was calculated using two-sided Mann-Whitney U-test with Benjamini–Hochberg correction.

SUPPLEMENTARY TABLES

Supplementary Table 1. dPLA performance for IL-6 and TNF-α in various buffers

dPLA Assays Target	dPLA Assays Media	Assay LOD [a] pg/ml (aM)	Sample LOD [b] pg/ml (fM)	Sample Sensitivity[c] pg/ml/DNA (molecule/DNA[d])
IL-6	BALF	1.38×10^{-4} (6.56)	1.53×10^{-2} (0.73)	9.99×10^{-3} (286.51)
	SDB-II[e]	7.79×10^{-4} (37.09)	8.65×10^{-2} (4.12)	2.02×10^{-3} (57.93)
TNF-α	BALF	6.69×10^{-4} (38.21)	7.58×10^{-2} (4.33)	4.07×10^{-4} (14.01)
	HMB[f]	7.07×10^{-4} (40.40)	8.01×10^{-2} (4.58)	7.36×10^{-3} (253.13)

[a] ALOD represents the limit of detection (LOD) in the assay reaction mixture. LOD is defined as analyte concentration at which the signal is 2 standard deviations above the background signal. [b] Sample LOD (SLOD) is the LOD in the original sample. [c] Sensitivity is the inverse of the slope of the calibration curve (lower is better). The fold difference between SLOD and ALOD corresponds to the dilution factor, ~113. [d] The number of proteins corresponds to one DNA molecule in assay or sample solutions.Human plasma samples for [e] IL-6 and [f]TNF-α dPLA are diluted in these buffer systems.

Supplementary Table 2. IL-6 dPLA performance in chicken and human plasma

Source	Assay LOD[a] pg/ml (fM)	Sample LOD[b] pg/ml (fM)	Sample Sensitivity [c] pg/ml/DNA (molecule/DNA[d])
100% chicken plasma	$5.78x10^{-2}$ (0.27)	6.42 (305.73)	0.78 ($2.22x10^{4}$)
20% chicken plasma	$3.43x10^{-2}$ (0.16)	3.81 (181.42)	0.24 ($6.93x10^{3}$)
20% human plasma	$7.56x10^{-3}$ (0.36)	0.84 (40.00)	$6.92x10^{-2}$ ($1.80x10^{3}$)

[a] Assay LOD (ALOD) represents the limit of detection (LOD) in the assay reaction mixture. LOD is defined as analyte concentration at which the signal is 2 standard deviations above the background average. [b] Sensitivity is the inverse of the slope of the calibration curve. A lower value means a higher sensitivity. [c] Sample LOD (SLOD) is the LOD in the original sample. The fold difference between SLOD and ALOD corresponds to the dilution factor, ~113. [d] The number of proteins corresponds to one DNA molecule in assay or sample solutions. CP: Chicken Plasma. HP: Human Plasma

Supplementary Table 3. Comparison of IL-6 dPLA with ELISA kits

Firm	LOD (pg/ml)	Sensitivity* (pg/ml/1 unit response)	Dynamic range (pg/ml)	Time/8 samples (hours)	Sample volume (μL)	Price ($/96 samples)
Company 1	0.11	4.5	0.2-10	5.5	100	599
Company 2	<1	190	10.24-400	3	50	459
Company 3	0.03	2.85	0.08-5	1.33	50	550
Company 4	1.6	167	7.8-500	1.75	50	515
Company 5	<0.8	25	1.56-50	3.75	100	529
Company 6	4	290	7.8-500	Oervight+5	100	295
This Study	*0.09* (SLOD)	*0.002*	*0.09-25*	*2.5*	*2*	*596*

* Sensitivity (i.e. analytical sensitivity) of ELISA assays was calculated by using the calibration curves provided by the company.

Supplementary Table 4. Performance comparison of TNF-α dPLA with ELISA kits

Firm	LOD (pg/ml)	Sensitivity* (pg/ml/1 unit response)	Dynamic range (pg/ml)	Time/ 8 samples (hours)	Sample volume (μL)	Price ($/96 samples)
Company 1	5.5	600	15.6-1000	3.5-4.5	200	519
Company 2	1.7	300	15.6-1000	4-5.5	100	459
Company 3	<0.09	6.4	0.5-32	4.5	50	510
Company 4	14	300	31.25-2000	1.5	50	515
Company 5	2	371	7.8-500	Overnight+5	100	295
Company 6	0.09	15.3	0.5-32	3	50	715
This Study	*0.08* (SLOD)	*0.007*	*0.08->50*	*Overnight +1.5*	*2*	*596*

* Sensitivity (i.e. analytical sensitivity) of ELISA assays was calculated by using the calibration curves provided by the company.

Supplementary Table 5. The sequences of the primers, probes and gBlock DNA fragments used in the study.

Name	Sequence (5'-3')*	Description	Source
TEM_fwd	GCATCTTACGGATGGCATGA	*bla*TEM forward primer	*Roschanski et al., 2014* [4]
TEM_rev	GTCCTCCGATCGTTGTCAGAA	*bla*TEM reverse primer	
TEM_probe	CAGTGCTGCCATAACCATGAGTGA	*bla*TEM TaqMan probe	
Bac_fwd (P967F)	CAA CGC GAA GAA CCT TAC C	Universal forward primer for both GN and GP bacteria	*Wu at al., 2008* [5]
Bac_rev (P1194R)	ACG TCA TCC CCA CCT TCC	Universal reverse primer for both GN and GP bacteria	
GN_probe	/HEX/ACG ACA GCC/ZEN/ATG CAG CAC CT/3IABkFQ/	Gram negative probe	
GP_probe	/FAM/ACG ACA ACC/ZEN/ATG CAG CAC CTG/3IABkFQ/	Gram positive probe	
GP_gBlock	ATA TAT CAA CGC GAA GAA CCT TAC CAA ATC TTG ACA TCC TTT GAC AAC TCT AGA GAT AGA GCC TTC CCC TTC GGG GGA CAA AGT GAC AGG TGG TGC ATG GTT GTC GTC AGC TCG TGT CGT GAG ATG TTG GGT TAA GTC CCG CAA CGA GCG CAA CCC TTA AGC TTA GTT GCC ATC ATT AAG TTG GGC ACT CTA AGT TGA CTG CCG GTG ACA AAC CGG AGG AAG GTG GGG ATG ACG TAA TTA T	Gram positive gBlock DNA fragment	*This study*
GN_gBlock	CAA CGC GAA GAA CCT TAC CTG GTC TTG ACA TCC ACA GAA CTT TCC AGA GAT GGA TTG GTG CCT TCG GGA ACT GTG AGA CAG GTG CTG CAT GGC TGT CGT CAG CTC GTG TTG TGA AAT GTT GGG TTA AGT CCC GCA ACG AGC GCA ACC CTT ATC CTT TGT TGC CAG CGG TCC GGC CGG GAA CTC AAA GGA GAC TGC CAG TGA TAA ACT GGA GGA AGG TGG GGA TGA CGT	Gram negative gBlock DNA fragment	
TEM_gBlock	GTG CAA AAA AGC GGT TAG CTC CTT CGG TCC TCC GAT CGT TGT CAG AAG TAA GTT GGC CGC AGT GTT ATC ACT CAT GGT TAT GGC AGC ACT GCA TAA TTC TCT TAC TGT CAT GCC ATC CGT AAG ATG CTT TTC TGT GAC TGG TGA GTA CTC	*bla*TEM gene gBlock DNA fragment	

* *FAM, 6-carboxyfluorescein; 3IABkFQ, 3' Iowa Black FQ quencher; HEX, 6-carboxyhexachlorofluorescein, ZEN, internal quencher*

Supplementary Table 6. Baseline and Outcome Clinical Characteristics of Septic Shock Patients

Variable	Value (N=32)	
Demographics		
Age [IQR]	61	[51-71]
Female	11	34%
Race		
African American	18	56%
White	11	34%
Other	3	9%
Illness Severity		
APACHE II score [IQR]	27	[25-32]
SOFA score [IQR]	11	[9-13]
Respiratory failure	17	53%
Positive blood culture	10	31%
Infection Site		
Soft tissue/skin	5	16%
Urinary	8	25%
Pulmonary	12	38%
Intrabdominal	4	13%
Line-associated	2	6%
Unknown	6	19%
Outcome Characteristics		
Hospital length of stay (days) [IQR]	11.6	[5.8-20.5]

	ICU length of stay (days) [IQR]	4.7	[3.1-10.5]
	Duration of shock (days) [IQR]	2.3	[1.6-4.7]
	In-hospital mortality	12	38%
Initial Laboratory and Vital Signs*			
	WBC count, g/dL, initial [IQR]	9.9	[5.1-19.1]
	Lactate, mmol/L [IQR]	2.9	[1.7-5.6]
	Maximum heart rate [IQR]	126	[111-138]
	Minimum heart rate [IQR]	82	[66-104]
	Maximum mean arterial pressure, mmHg [IQR]	97.5	[111-138]
	Minimum mean arterial pressure, mmHg [IQR]	49	[46-56]
	Maximum temperature,°C [IQR]	37.2	[36.9-38.6]
	Minimum temperature,°C [IQR]	36.5	[36.1-36.6]

Abbreviations: APACHE, Acute Physiologic Assessment and Chronic Health Evaluation: IQR, interquartile range; SOFA, Sequential Organ Failure Assessment, *: Values represent the minimum and maximum values recorded in the first 24 hours of enrollment.

Supplementary Table 7. Comparision of Outcome Clinical Characteristics between Survivors and Nonsurvivors of Septic Shock Patients

	Survivors [IQR] (n=20)		Nonsurvivors [IQR] (n=12)		P value
Age	62.5	[50.6-72.4]	58.7	[53.6-76.5]	0.95
Apache II Score	25	[21-27.5]	29.5	[28-35]	0.002
SOFA Score	10.5	[7-11.5]	13	[0-15.5]	0.04
Respiratory Failure	8	40%	9	75%	0.05
WBD count, initial	8.5	[5.3-16.4]	16.9	[2.6-23.5]	0.3
Lactate, initial	2.65	[1.65-4]	5.5	[1.7-8.75]	0.11
24 hour Lactate Clearance, %	47.5	[-7.7-68.7]	1.6	[-1.6-33.7]	0.13

Two-sided Wilcoxon-Mann-Whitney two-sample ranksum test to compare continuous variables and the chi-squared test or Fisher exact test where appropriate to compare categorical variables.

Supplementary References

1. Somers, W., Stahl, M. & Seehra, J.S. 1.9 A crystal structure of interleukin 6: implications for a novel mode of receptor dimerization and signaling. *The EMBO journal* **16**, 989-997 (1997).
2. Albayrak, C.*, et al.* Digital quantification of proteins and mRNA in single mammalian cells. *Molecular cell* **61**, 914-924 (2016).
3. Fernando, S.A. & Wilson, G.S. Studies of the 'hook'effect in the one-step sandwich immunoassay. *Journal of immunological methods* **151**, 47-66 (1992).
4. Roschanski, N., Fischer, J., Guerra, B. & Roesler, U. Development of a multiplex real-time PCR for the rapid detection of the predominant beta-lactamase genes CTX-M, SHV, TEM and CIT-type AmpCs in Enterobacteriaceae. *PloS one* **9**, e100956 (2014).
5. Wu, Y.D.*, et al.* Gram stain-specific-probe-based real-time PCR for diagnosis and discrimination of bacterial neonatal sepsis. *J Clin Microbiol* **46**, 2613-2619 (2008).
