# Supplementary material for: Ultrasensitive digital quantification of cytokines and bacteria predicts septic shock outcomes
Source: Nat Commun. 2020 May 25;11:2607. doi: 10.1038/s41467-020-16124-9 (PMC7248118; doi:10.1038/s41467-020-16124-9)
Supplement: Supplementary file 2 — Description of Additional Supplementary Files [file 41467_2020_16124_MOESM2_ESM.pdf]

Description of Additional Supplementary Files

File Name: Supplementary Software 1
Description: The R code used to perform the two-sided two-part statistics test.
