# Supplementary material for: Ultrasensitive digital quantification of cytokines and bacteria predicts septic shock outcomes
Source: Nat Commun. 2020 May 25;11:2607. doi: 10.1038/s41467-020-16124-9 (PMC7248118; doi:10.1038/s41467-020-16124-9)
Supplement: Supplementary file 4 — Reporting Summary [file 41467_2020_16124_MOESM4_ESM.pdf]

nature research

Corresponding author(s): SAVAS TAY, BHAKTI PATEL

Last updated by author(s): 28/3/2020

Reporting Summary

Nature Research wishes to improve the reproducibility of the work that we publish. This form provides structure for consistency and transparency in reporting. For further information on Nature Research policies, see Authors & Referees and the Editorial Policy Checklist.

Statistics

For all statistical analyses, confirm that the following items are present in the figure legend, table legend, main text, or Methods section.

n/a	Confirmed	
☐	☒	The exact sample size (*n*) for each experimental group/condition, given as a discrete number and unit of measurement
☐	☒	A statement on whether measurements were taken from distinct samples or whether the same sample was measured repeatedly
☐	☒	The statistical test(s) used AND whether they are one- or two-sided<br>*Only common tests should be described solely by name; describe more complex techniques in the Methods section.*
☒	☐	A description of all covariates tested
☐	☒	A description of any assumptions or corrections, such as tests of normality and adjustment for multiple comparisons
☐	☒	A full description of the statistical parameters including central tendency (e.g. means) or other basic estimates (e.g. regression coefficient) AND variation (e.g. standard deviation) or associated estimates of uncertainty (e.g. confidence intervals)
☐	☒	For null hypothesis testing, the test statistic (e.g. *F*, *t*, *r*) with confidence intervals, effect sizes, degrees of freedom and *P* value noted<br>*Give P values as exact values whenever suitable.*
☒	☐	For Bayesian analysis, information on the choice of priors and Markov chain Monte Carlo settings
☒	☐	For hierarchical and complex designs, identification of the appropriate level for tests and full reporting of outcomes
☒	☐	Estimates of effect sizes (e.g. Cohen's *d*, Pearson's *r*), indicating how they were calculated

Our web collection on statistics for biologists contains articles on many of the points above.

Software and code

Policy information about availability of computer code

Data collection	QuantaSoft Software from Bio-Rad (ddPCR data collection), Bio-Rad CFX Maestro. QuantaSoft Analysis Pro software (Bio-Rad, v.1.0.596), Bio-Rad CFX Maestro 1.1 (Bio-Rad, v.4.1.2433.1219).
Data analysis	Customized codes written with R and Python. Code file (twopart_statistics.R) is included in the manuscript as additional supplementary files.

For manuscripts utilizing custom algorithms or software that are central to the research but not yet described in published literature, software must be made available to editors/reviewers. We strongly encourage code deposition in a community repository (e.g. GitHub). See the Nature Research guidelines for submitting code & software for further information.

Data

Policy information about availability of data

All manuscripts must include a data availability statement. This statement should provide the following information, where applicable:

- Accession codes, unique identifiers, or web links for publicly available datasets
- A list of figures that have associated raw data
- A description of any restrictions on data availability

The datasets generated during and/or analyzed during the current study are available from the corresponding author on reasonable request.

Field-specific reporting

Please select the one below that is the best fit for your research. If you are not sure, read the appropriate sections before making your selection.

☒ Life sciences ☐ Behavioural & social sciences ☐ Ecological, evolutionary & environmental sciences

October 2018

Life sciences study design

All studies must disclose on these points even when the disclosure is negative.

Sample size	Sample size was not determined beforehand. The number of patients enrolled in our study and the number of samples collected dependent on patient availability, clinical outlook, and patient consent, and thus were not determined beforehand.
Data exclusions	qPCR measurements were done in triplicates, some qPCR data were excluded if they were outliers. Exclusion of extreme outliers is standard practice in qPCR measurements and most likely represent technical errors (i.e. PCR reaction not being completed)
Replication	During the preparation of DNA samples for optimization of ddPCR experiment, for calibration curves with appropriate DNA standards, each standard concentration was measured in triplicates at ddPCR/qPCR level. Each patient sample was measured in duplicate.<br>For simultaneous quantification of GN and GP bacteria and GN, GP and blaTEM genes experiments, each standard concentration was measured in triplicates at ddPCR level, each patient sample was measured in duplicate.<br>For simultaneous quantification of IL-6 and DNA targets, each standard concentration was measured in triplicates at ddPCR level, each patient sample was measured in duplicate.<br>For Multiplexed Lumined assay, each standard concentration was measured in triplicates at ddPCR level, each patient sample was measured in duplicate.<br>For Quantification of bacterial DNA targets and host cytokines experiment, each standard concentration and patient sample were measured in triplicates at ddPCR level.<br>The number of replication (n values, >3) for each experiment are directly included in the text or figures. Attempts at replication all experiments were successful.
Randomization	There was no randomization for this study. All samples were collected from patients as part of a prospective trial and stored in a biorepository for later analysis. There was no intervention performed that required randomization.
Blinding	Blinding is not relevant in this prospective biomarker study. The clinical characteristics and phenotypes of subjects were not known to the researchers performing the digital assay at the time of processing.

Reporting for specific materials, systems and methods

We require information from authors about some types of materials, experimental systems and methods used in many studies. Here, indicate whether each material, system or method listed is relevant to your study. If you are not sure if a list item applies to your research, read the appropriate section before selecting a response.

Materials & experimental systems

n/a	Involved in the study
☐	☒ Antibodies
☒	☐ Eukaryotic cell lines
☒	☐ Palaeontology
☒	☐ Animals and other organisms
☐	☒ Human research participants
☒	☐ Clinical data

Methods

n/a	Involved in the study
☒	☐ ChIP-seq
☒	☐ Flow cytometry
☒	☐ MRI-based neuroimaging

Antibodies

Antibodies used	anti-IL-6 (R&D Systems, Cat #: BAF206), anti-TNFalpha (R&D Systems, Cat #: BAF210). The biotinylated antibodies were diluted to a concentration of 200 nM with antibody dilution buffer (ADB) (Thermo Fisher Scientific, #4448571).
Validation	We validated the antibody specificity with dPLA ourselves (see Figure 1d).

Human research participants

Policy information about studies involving human research participants

Population characteristics	The number of patients with septic shock studied in this manuscript is 32 (age [IQR]: 61[51-71]; female: 34%; the major race groups: African-American (56%), White (34%), Others (9%); APACHE II score [IQR]: 27 [25-32]; SOFA score [IQR]: 11 [9-13]; Respiratory failure: 53%; Positive blood culture: 31%). Patients were diagnosed with septic shock based on the criteria established by the Sepsis-3 guidelines. Briefly, all adult patients > 18 years of age were initially admitted to the Medical Intensive Care Unit at the University of Chicago. These patients who required vasoactive medication support for blood pressure and who had a suspected infection were approached for enrollment within the first 24 hours of diagnosis. Because the standard of care at our institution is to administer antibiotics within 3 hours of sepsis suspicion, all patients had received antibiotic therapy prior to sample collection.<br>All BALF study subjects we recruited have provided written and informed consent for participation. To provide a broad range of

disease states, we recruited 23 subjects with different stages of asthma. These patients included those who have mild disease (no use of inhaled or oral corticosteroid), patients that required inhaled corticosteroid for disease control, and patients who required oral corticosteroids for disease control. Subjects withheld the use of long-acting beta-adrenergic agonists for 24 hours prior to the first visit, at which time medical history, medication use, and asthma questionnaires were administered. Subjects recruited in this study met the criteria defined by the EPR 3 Guidelines on Asthma. Eleven control subjects we also recruited and they had no lifetime history of pulmonary disease, they were in good health, did not use respiratory-related medication. Control subjects showed <16% reduction in FEV1 after inhalation of 25 mg/ml methacholine. Subjects who were excluded include those who had a smoking history of ≥10 pack/years, who within 6 months of recruitment were actively smoking, who had a history of allergic bronchopulmonary aspergillosis, chronic obstructive pulmonary disease, Churg-Strauss syndrome, or had any contraindication to bronchoscopy. For asthmatic patients, average age:46 [38-53], 53% female, 65% white, 30% black and 4% others.

Recruitment

Patients aged ≥18 years of age with shock admitted to the medical intensive care unit at the University of Chicago were eligible for enrollment. There were no exclusion criteria. If eligible, patients or their surrogates were approached within the first 24 hours of diagnosis for informed consent. Patients were enrolled in the study if consent was obtained.
All BALF subjects were recruited by local advertising or through clinical referral for diagnosis of asthma into an NIH-sponsored study that examined the role of the innate immunity factor HLA-G in asthma (AI-095230, S. White, contact PI). This was an observational study that did not require randomization and the approval of the University of Chicago Institutional Review Board specifically permitted the use of remaining samples in new studies. No self-selection or other biases were applied to the selection of patients beyond what is described above (there were no exclusion criteria for septic shock patients, exclusion criteria for asthma patients is described above, under population characteristics). For asthmatic patients, average age:46 [38-53], 53% female, 65% white, 30% black and 4% others.

Ethics oversight

The index study (noted in Recruitment) was approved by the University of Chicago Institutional Review Board; the IRB specifically permitted the use of remaining samples in new studies. The index study had oversight from NIAID program staff throughout the time the grant was active.

Note that full information on the approval of the study protocol must also be provided in the manuscript.
